# Waning effectiveness against COVID-19-related hospitalization, severe complications, and mortality with two to three doses of CoronaVac and BNT162b2: a case–control study

**DOI:** 10.1080/22221751.2023.2209201

**Published:** 2023-05-18

**Authors:** Vincent Ka Chun Yan, Eric Yuk Fai Wan, Xuxiao Ye, Anna Hoi Ying Mok, Francisco Tsz Tsun Lai, Celine Sze Ling Chui, Xue Li, Carlos King Ho Wong, Philip Hei Li, Tiantian Ma, Simon Qin, Chak Sing Lau, Ian Chi Kei Wong, Esther Wai Yin Chan

**Affiliations:** a Department of Pharmacology and Pharmacy, Li Ka Shing Faculty of Medicine, Centre for Safe Medication Practice and Research, The University of Hong Kong, Hong Kong, People’s Republic of China; b Laboratory of Data Discovery for Health (D^2^4H), Hong Kong Science and Technology Park, Hong Kong, People’s Republic of China; c Department of Family Medicine and Primary Care, School of Clinical Medicine, Li Ka Shing Faculty of Medicine, The University of Hong Kong, Hong Kong, People’s Republic of China; d School of Nursing, Li Ka Shing Faculty of Medicine, The University of Hong Kong, Hong Kong, People’s Republic of China; e School of Public Health, Li Ka Shing Faculty of Medicine, The University of Hong Kong, Hong Kong, People’s Republic of China; f Department of Medicine, School of Clinical Medicine, Li Ka Shing Faculty of Medicine, The University of Hong Kong, Hong Kong, People’s Republic of China; g Research Department of Practice and Policy, School of Pharmacy, University College London, London, UK; h Aston Pharmacy School, Aston University, Birmingham, UK; i Department of Pharmacy, The University of Hong Kong-Shenzhen Hospital, Shenzhen, People’s Republic of China; j The University of Hong Kong Shenzhen Institute of Research and Innovation, Shenzhen, People’s Republic of China

**Keywords:** COVID-19, waning vaccine effectiveness, CoronaVac, BNT162b2, Omicron

## Abstract

Background: This study aims to evaluate waning effectiveness against severe and fatal COVID-19 with two to three doses of CoronaVac/BNT162b2, where data are limited. Methods: A case–control study included individuals aged ≥18 years, unvaccinated or received two to three doses of CoronaVac/BNT162b2, using electronic healthcare databases in Hong Kong. Those with first COVID-19-related hospitalization, severe complications, or mortality between 1 January and 15 August 2022 were defined as cases and matched with up-to-10 controls by age, sex, index date, and Charlson Comorbidity Index. Vaccine effectiveness (VE) against COVID-19-related outcomes was estimated at different time intervals from second and third-dose vaccination (0–13 up-to 210–240 days) using conditional logistic regression adjusted for comorbidities and medications. Results: By 211–240 days after second dose, VE against COVID-19-related hospitalization reduced to 46.6% (40.7–51.8%) for BNT162b2 and 36.2% (28.0–43.4%) for CoronaVac, and VE against COVID-19-related mortality were 73.8% (55.9–84.4%) and 76.6% (60.8–86.0%). After third dose, VE against COVID-19-related hospitalization decreased from 91.2% (89.5–92.6%) for BNT162b2 and 76.7% (73.7–79.4%) for CoronaVac at 0–13 days, to 67.1% (60.4–72.6%) and 51.3% (44.2–57.5%) at 91–120 days. VE against COVID-19-related mortality for BNT162b2 remained high from 0–13 days [98.2% (95.0–99.3%)] to 91–120 days [94.6% (77.7–98.7%)], and for CoronaVac reduced from 0–13 days [96.7% (93.2–98.4%)] to 91–120 days [86.4% (73.3–93.1%)]. Conclusions: Significant risk reduction against COVID-19-related hospitalization and mortality after CoronaVac or BNT162b2 vaccination was observed for >240 and >120 days after second and third doses compared to unvaccinated, despite significant waning over time. Timely administration of booster doses could provide higher levels of protection.

## Introduction

The Omicron (B.1.1.529) variant of severe acute respiratory syndrome coronavirus 2 (SARS-CoV-2) has become the dominant strain of Coronavirus disease (COVID-19) that swept the globe. Many countries, such as China, Singapore and Australia, have achieved vaccination rates over 80% for the primary series of COVID-19 vaccine [1], but the number of confirmed cases worldwide still surged during the Omicron outbreak. In Hong Kong, after more than 6 months of almost zero new cases of COVID-19 and related deaths in 2021, Hong Kong faced a major outbreak with more than 50,000 new cases and close to 300 deaths recorded daily at its peak in early March 2022 [2], which have continued to place a significant burden on the healthcare system to date.

COVID-19 vaccines of two major vaccine platforms, namely BNT162b2 from Fosun-BioNTech (equivalent to Pfizer-BioNTech, mRNA vaccine) and CoronaVac from Sinovac Biotech (HK) Limited (inactivated vaccine) have been available in Hong Kong for individuals aged ≥16 years since 23 February 2021 for CoronaVac and aged ≥18 years since 6 March 2021 for BNT162b2. COVID-19 booster shots were made available to priority groups on 11 November 2021, and subsequently expanded to the general population from 1 January 2022. Individuals have a choice between BNT162b2 or CoronaVac for their first dose and are restricted to the same vaccine for their second dose. For the booster shot, either a homologous or heterologous booster was permitted. Since the launch of mass vaccination program in many countries in 2021, it has been more than a year after the completion of the primary series in those who started vaccination earliest. Nonetheless, the coverage of the booster dose lags far behind that of the primary series, resulting in a population susceptible to COVID-19 infection as protection offered by the primary series might have waned over time.

The phenomenon of waning immunity after natural infection or COVID-19 vaccination has been well described in previous studies. A Chinese study reported that the IgG antibody level in COVID-19 convalescent plasma declined with time to around 35.7% of individuals’ baseline by 9 months [3]. In Qatar, the estimated effectiveness of BNT162b2 (Pfizer–BioNTech) vaccine against hospitalization and death dropped from 96.0% (93.9–97.4) to an insignificant value 6 months after the second dose during an outbreak when Delta was the dominant variant [4]. Meanwhile, the United Kingdom (UK) recorded a vaccine effectiveness (VE) of 91.9% (88.5–94.3) against Delta variant-related death by 20 weeks after the second dose of BNT162b2 [5]. The extent of the decline in terms of the protection offered by the third dose is less explored. In Israel, VE of BNT162b2 against infection decreased from 53.4% (47.7–58.6) to 16.5% (13–19.9) after three months since third dose vaccination during the Omicron wave [6], but the decline in VE against hospitalizations or deaths was not found to be significant because of a small number of outcome events recorded by the end of 2021 [6]. In South Africa, VE of the third dose of BNT162b2 against hospitalization declined to 50% (4.4–73.9) during the BA.1–BA.2 outbreak after 3–4 months [7], representing a large discrepancy in VE across different countries. Estimates for waning VE other than BNT162b2, such as inactivated vaccines, remain limited. In view of the rise of newer variants or sub-lineages, whether three doses of vaccination provide adequate protection remains uncertain. This information may be useful in determining the optimal timing of a third or even fourth dose to boost protection against severe COVID-19.

Despite being the most widely used COVID-19 vaccine globally [8], CoronaVac has been scarcely studied in terms of the effect of waning immunity. A serological study reported a substantial decrease in IgG seropositivity in Chileans who received two doses of CoronaVac [9], but this does not necessarily equate to a decline in real-life effectiveness as immunity against SARS-CoV-2 is not solely contributed by neutralizing antibodies [10]. It is in our interest to evaluate the extent of the decline in terms of VE against severe and fatal outcomes. Considering the high COVID-19 death rate observed locally [11], this study aims to examine the phenomenon of waning effectiveness of BNT162b2 and CoronaVac against COVID-19-related hospitalization, severe complications and mortality during the Omicron-dominant outbreak in HK where the coverage of booster doses is suboptimal.

## Methods

### Study design and population

This is a case–control study conducted among individuals aged ≥18 years. Routine electronic health records were extracted from the clinical management system (CMS) under the Hospital Authority (HA) of Hong Kong. The CMS manages data on demographics, diagnoses, prescriptions, and laboratory tests, and provides real-time data support and monitoring for routine clinical management across all clinics and hospitals in HA. Individuals who had received either none or at least two doses of vaccinations between 1 January 2022 and 15 August 2022 were identified and included in the cohort. Vaccination records were extracted from the Department of Health (DH) of the Government of the Hong Kong Special Administrative Region (HKSAR). The DH manages and retains the database for all vaccination records in Hong Kong. The Centre for Health Protection (CHP) maintains a database of all confirmed COVID-19 cases, based on both mandatory and voluntary reporting of positive Rapid Antigen Test (RAT) and Polymerase Chain Reaction (PCR) test results. These databases are linked based on unique personal identifiers and have been used previously to conduct studies on the risk of adverse effects after COVID-19 vaccinations and other COVID-19 pharmacovigilance studies [12–20].

To evaluate waning in VE after two or three doses of COVID-19 vaccines against outcomes after contracting predominantly Omicron variant [21], the inclusion period for each outcome ranged from 1 January 2022 to 15 August 2022. Trends of new COVID-19 cases during the study period were presented in Supplementary Figure 1. Those who had a previous COVID-19 infection before the index date, or had received only one dose or the fourth dose of COVID-19 vaccine were excluded from the analysis. Due to the limited proportion of individuals receiving a heterologous booster since the commencement of the booster dose mass vaccination, these individuals were also excluded from the analysis.

### Definitions of vaccine exposure

Time since vaccination was defined as index date minus the date of vaccination of the latest COVID-19 vaccine dose. To estimate VE against each outcome of the second dose of BNT162b2 or CoronaVac, nine time since-vaccination intervals (0–13, 14–30, 31–60, 61–90, 91–120, 121–150, 151–180, 181–210 and 210–240 days) were investigated; whilst VE of the third dose of vaccination was estimated for seven time since-vaccination intervals (0–13, 14–30, 31–60, 61–90, 91–120, 121–150, and 151–180 days). Previous studies suggested that the vaccines elicit full immune response in most patients by 14 days after receiving the second dose [22,23], thus we investigated vaccine effectiveness for the 0–13 days interval in addition to the subsequent monthly intervals. Those who did not receive any COVID-19 vaccine before the index date were considered unvaccinated.

### Definitions of COVID-19-related outcomes

The outcomes investigated in this study were (i) COVID-19-related hospitalization, (ii) COVID-19-related mortality, and (iii) COVID-19-related severe complications. COVID-19-related hospitalization was defined as hospital admission within 28 days after a PCR-confirmed COVID-19 infection. COVID-19-related mortality was defined as all-cause mortality within 28 days after a PCR-confirmed COVID-19 infection. All-cause mortality data were based on the Hong Kong Deaths Registry, which officially records all registered deaths of Hong Kong residents. COVID-19-related severe complications were defined as the admission to the intensive care unit (ICU) or use of ventilatory support within 28 days after a PCR-confirmed COVID-19 infection. Use of ventilatory support, including intubation, mechanical ventilation, and oxygen supplementation, identified using the International Classification of Diseases, Ninth Revision, clinical modification (ICD-9-CM) procedure codes (39.65, 89.18, 93.90, 93.95, 93.96, 96.04, 96.7×). COVID-19 infection was defined as a positive polymerase chain reaction (PCR) test confirmed by the Centre of Health Protection of the HKSAR government. PCR test results were recognized as the gold standard diagnostic criteria for COVID-19 infection given its high specificity of >99% [24]. The Hong Kong government has implemented extensive PCR testing for SARS-CoV-2 in public hospitals and clinics for close contacts of confirmed cases and those who presented with COVID-like symptoms. The government also set up territory-wide community testing centres to screen asymptomatic individuals and provide regular testing to various staff groups with a high risk of exposure to COVID-19, such as those working in nursing homes.

### Statistical analysis

Case and control matching was conducted separately for each outcome. Patients with the outcome of interest during the inclusion period were included as cases; while all other patients with attendance to any HA health services (i.e. hospital admissions, emergency departments, and outpatient clinics) but without the outcome of interest were selected as controls. Up to ten controls were randomly matched with the cases according to sex, age (5-year band), date of attendance (within three calendar days), and Charlson Comorbidity Index (0, 1–2, 3–4, ≥5) [25].

For each time since-vaccination interval, only eligible matched pairs, in which both the case and controls were either unvaccinated or fell within the specific time since-vaccination interval, were included to derive the corresponding estimates. Conditional logistic regression adjusted for chronic comorbidities (cancer, chronic kidney disease, respiratory disease, diabetes mellitus, cardiovascular disease, dementia), and the use of chronic medications (renin-angiotensin-system agents, beta-blockers, calcium channel blockers, diuretics, nitrates, lipid-lowering agents, insulins, antidiabetic drugs, oral anticoagulants, antiplatelets, immunosuppressants) was used to evaluate the association between vaccination and the risk of COVID-19-related outcome. Vaccine effectiveness (VE) was estimated by (1 - adjusted OR) × 100%. Subgroup analyses stratified by age (<65; ≥65 years), sex (male; female), and Charlson Comorbidity Index (<2; ≥2) were conducted. Simple linear regression on the VE point estimates was also used to test the linear trend in rate of change of VE after second or third-dose vaccination (Supplementary Figure 2).

**Figure 1.**
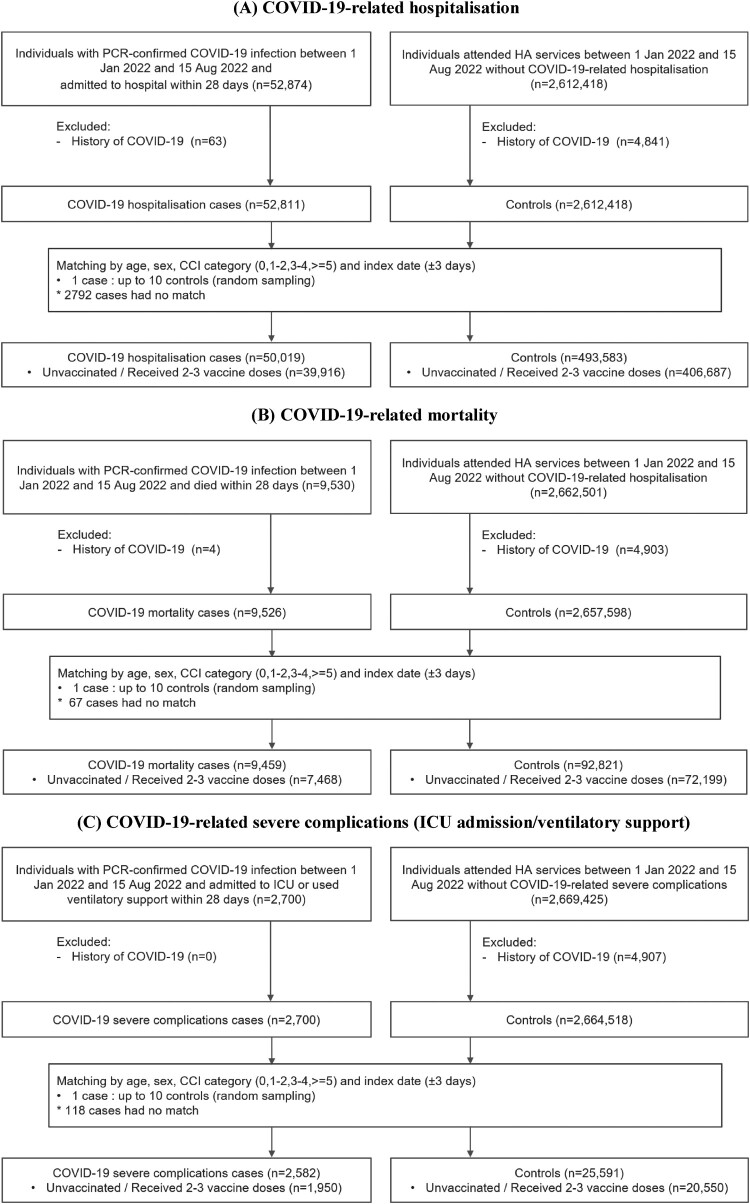
Selection of cases and controls. (A) COVID-19-related hospitalization; (B) COVID-19-related mortality; COVID-19-related severe complications (ICU admission/ventilatory support).

**Figure 2.**
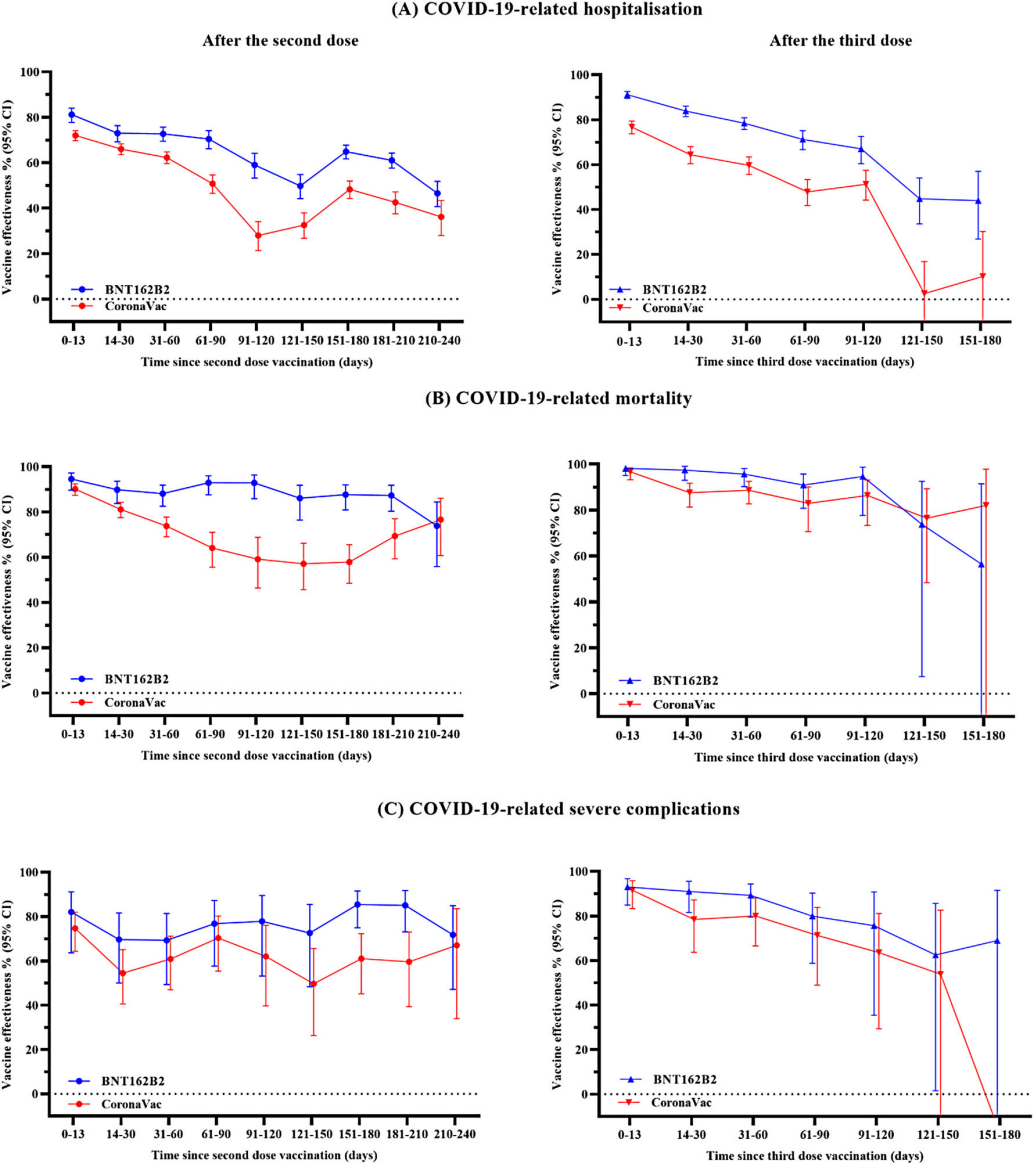
Vaccine effectiveness against COVID-19 outcomes over different time intervals after COVID-19 vaccination. This figure shows the vaccine effectiveness against COVID-19 outcomes over different time intervals after vaccination of BNT162b2 and CoronaVac. Results presented in this figure should not be interpreted as a direct comparison of effectiveness of the two vaccines.

All statistical tests were two-sided, and *P* values less than 0.05 were considered statistically significant. Statistical analysis was conducted using R version 4.0.3 (www.R-project.org). At least two investigators (VY, EW) conducted the statistical analyses independently for quality assurance. STROBE (Strengthening the Reporting of Observational Studies in Epidemiology) statement checklists were followed to guide transparent reporting of the case–control study [20].

### Ethics approval

This study was approved by the Central Institutional Review Board of the Hospital Authority of Hong Kong (CIRB-2021-005-4) and the DH Ethics Committee (LM171/2021). Informed consent was waived by the ethics committee since this study only uses anonymized patient data.

## Results

A total of 39,916 cases of COVID-19-related hospitalization, 7468 cases of COVID-19-related mortality, and 1950 COVID-19-related severe complications were matched with 406,687; 72,199 and 20,550 controls, respectively (Figure 1). Baseline characteristics of cases and controls are summarized in Table 1. Time trends of new COVID-19 cases, hospitalization, mortality and severe complications during the study period are presented in Supplementary Figure 1.

**Table 1.** Baseline characteristics of cases and controls.

	COVID-19-related hospitalization		COVID-19-related mortality		COVID-19-related severe complications	
	Case	Control	Case	Control	Case	Control
Number of individuals	39916	406687	7468	72199	1950	20550
Age, years – mean (SD)	70.62 (19.53)	70.57 (19.22)	83.94 (11.51)	83.42 (11.32)	73.67 (15.67)	73.44 (15.33)
Sex, male (%)	20347 (51.0)	209018 (51.4)	4351 (58.3)	42611 (59.0)	1205 (61.8)	12603 (61.3)
Charlson Comorbidity Index – mean (SD)	1.37 (1.88)	1.22 (1.74)	2.21 (2.21)	1.97 (2.05)	1.53 (1.84)	1.38 (1.79)
Time since recent dose – mean (SD)	114.42 (82.40)	93.18 (81.00)	105.07 (82.29)	68.03 (68.51)	94.90 (76.44)	84.76 (77.51)
**Comorbidities – no. (%)**						
Cancer	4333 (10.9)	35803 (8.8)	971 (13.0)	9376 (13.0)	156 (8.0)	1992 (9.7)
Chronic Kidney Disease	3492 (8.7)	29924 (7.4)	1138 (15.2)	10333 (14.3)	271 (13.9)	1762 (8.6)
Respiratory disease	3320 (8.3)	25529 (6.3)	965 (12.9)	7531 (10.4)	228 (11.7)	1393 (6.8)
Diabetes	9838 (24.6)	130050 (32.0)	2337 (31.3)	30676 (42.5)	581 (29.8)	7600 (37.0)
Cardiovascular disease	20987 (52.6)	233322 (57.4)	5404 (72.4)	55082 (76.3)	1128 (57.8)	13125 (63.9)
Dementia	1863 (4.7)	7825 (1.9)	806 (10.8)	2999 (4.2)	90 (4.6)	477 (2.3)
**Medications within past 90 days – no. (%)**						
Renin-angiotensin-system agents	11937 (29.9)	132903 (32.7)	2619 (35.1)	30889 (42.8)	723 (37.1)	7641 (37.2)
Beta-blockers	8956 (22.4)	80571 (19.8)	2267 (30.4)	18545 (25.7)	538 (27.6)	4699 (22.9)
Calcium channel blockers	15822 (39.6)	176974 (43.5)	3893 (52.1)	40877 (56.6)	891 (45.7)	9928 (48.3)
Diuretics	6980 (17.5)	35692 (8.8)	2708 (36.3)	10439 (14.5)	506 (25.9)	2038 (9.9)
Nitrates	3596 (9.0)	23496 (5.8)	1116 (14.9)	6555 (9.1)	231 (11.8)	1360 (6.6)
Lipid-lowering agents	15339 (38.4)	183991 (45.2)	3280 (43.9)	41495 (57.5)	855 (43.8)	10729 (52.2)
Insulins	3342 (8.4)	17686 (4.3)	2055 (27.5)	4398 (6.1)	267 (13.7)	1148 (5.6)
Antidiabetic drugs	8568 (21.5)	109652 (27.0)	1812 (24.3)	24365 (33.7)	529 (27.1)	6510 (31.7)
Oral anticoagulants	2535 (6.4)	16075 (4.0)	702 (9.4)	4737 (6.6)	151 (7.7)	908 (4.4)
Antiplatelets	11004 (27.6)	91050 (22.4)	3239 (43.4)	24277 (33.6)	628 (32.2)	5271 (25.6)
Immunosuppressants	824 (2.1)	2362 (0.6)	451 (6.0)	298 (0.4)	93 (4.8)	142 (0.7)
Antibacterial drugs (within 7 days)	6751 (16.9)	2258 (0.6)	5970 (79.9)	910 (1.3)	456 (23.4)	202 (1.0)
Antiviral drugs (within 7 days)	3099 (7.8)	7845 (1.9)	1162 (15.6)	1764 (2.4)	168 (8.6)	512 (2.5)

A significant risk reduction against COVID-19-related hospitalization with CoronaVac or BNT162b2 was observed for at least 240 days after the second dose and 120 days after the third dose when compared to the unvaccinated individuals, despite significant waning in VE (Figure 2, Tables 2 and 3). VE (95% CI) against COVID-19-related hospitalization decreased from 81.1% (77.7–84.1%) for BNT162b2 and 72.0% (69.7–74.1%) for CoronaVac at 0–13 days, to 46.6% (40.7–51.8%) for BNT162b2 and 36.2% (28.0–43.4%) for CoronaVac at 211–240 days after the second dose. For COVID-19-related mortality, VE for BNT162b2 remained consistently high from 0–13 days [94.6% (89.6–97.1%)] to 181–210 days [87.3% (80.2–91.8%)] after the second dose, and reduced to 73.8% (55.9–84.4%) by 211–240 days, whereas VE for CoronaVac waned gradually from 0–13 days [90.2% (87.3–92.3%)] to 211–240 days [76.6% (60.8–86.0%)] after the second dose. No significant waning of VE for COVID-19-related severe complications was observed, where VE was 82.1% (63.7–91.1%) for BNT162b2 and 74.7% (64.3–82.0%) for CoronaVac at 0–13 days, and 71.8% (47.2–84.9%) for BNT162b2 and 67.1% (34.0–83.6%) for CoronaVac at 211–240 days after the second dose.

**Table 2.** Vaccine effectiveness against COVID-19 outcomes over different time intervals after second-dose COVID-19 vaccination.

Days since 2nd dose	0–13	14–30	31–60	61–90	91–120	121–150	151–180	181–210	211–240
COVID-19-related hospitalisation									
**BNT162b2**									
Case (*n*_u_/*n*_v_)	18147/160	18255/269	18372/390	18067/269	17912/293	18003/509	18365/761	18381/823	17985/567
Control (*n*_u_/*n*_v_)	58603/2408	59351/2848	59722/3739	58558/2281	58396/1886	59180/2736	59827/5562	59204/5094	57872/2348
VE (95% CI)	81.1 (77.7–84.1)	73.0 (69.2–76.3)	72.7 (69.5–75.6)	70.4 (66.1–74.1)	59.0 (53.2–64.1)	49.8 (44.2–54.8)	64.8 (61.7–67.7)	61.0 (57.6–64.2)	46.6 (40.7–51.8)
**CoronaVac**									
Case (*n*_u_/*n*_v_)	18147/792	18255/1065	18372/1250	18067/877	17912/790	18003/864	18365/1070	18381/858	17985/419
Control (*n*_u_/*n*_v_)	58603/8980	59351/9833	59722/9456	58558/4706	58396/3019	59180/3695	59827/5679	59204/3867	57872/1448
VE (95% CI)	72.0 (69.7–74.1)	66.0 (63.5–68.3)	62.2 (59.6–64.7)	50.8 (46.5–54.7)	28.0 (21.4–34.0)	32.6 (26.8–37.9)	48.3 (44.3–51.9)	42.5 (37.6–47.1)	36.2 (28.0–43.4)
COVID-19-related mortality									
**BNT162b2**									
Case (*n*_u_/*n*_v_)	5800/11	5794/20	5838/33	5744/15	5706/11	5706/17	5758/27	5772/29	5708/21
Control (*n*_u_/*n*_v_)	20965/671	21242/794	21343/1049	21126/601	20974/379	21074/425	21238/697	20958/610	20803/233
VE (95% CI)	94.6 (89.6–97.1)	89.8 (83.7–93.6)	88.1 (82.5–91.8)	92.9 (87.6–96.0)	92.8 (85.8–96.4)	86.1 (76.4–91.8)	87.7 (80.9–92.0)	87.3 (80.2–91.8)	73.8 (55.9–84.4)
**CoronaVac**									
Case (*n*_u_/*n*_v_)	5800/68	5794/159	5838/195	5744/128	5706/79	5706/102	5758/142	5772/71	5708/19
Control (*n*_u_/*n*_v_)	20965/2980	21242/3291	21343/2929	21126/1242	20974/729	21074/868	21238/1180	20958/758	20803/247
VE (95% CI)	90.2 (87.3–92.3)	81.1 (77.4–84.2)	73.7 (69.0–77.7)	64.0 (55.5–70.9)	59.1 (46.4–68.7)	57.1 (45.5–66.2)	57.8 (48.4–65.5)	69.3 (59.2–77.0)	76.6 (60.8–86.0)
COVID-19-related severe complications									
**BNT162b2**									
Case (*n*_u_/*n*_v_)	1039/10	1046/21	1051/20	1042/13	1033/8	1032/12	1058/17	1056/16	1027/14
Control (*n*_u_/*n*_v_)	3354/130	3476/193	3421/204	3321/142	3320/109	3349/110	3365/268	3324/220	3273/100
VE (95% CI)	82.1 (63.7–91.1)	69.7 (50.0–81.6)	69.3 (49.4–81.4)	76.8 (57.7–87.3)	77.8 (53.2–89.5)	72.6 (48.4–85.4)	85.5 (74.9–91.6)	85.1 (73.2–91.7)	71.8 (47.2–84.9)
**CoronaVac**									
Case (*n*_u_/*n*_v_)	1039/44	1046/83	1051/64	1042/35	1033/26	1032/39	1058/53	1056/38	1027/11
Control (*n*_u_/*n*_v_)	3354/522	3476/571	3421/484	3321/259	3320/179	3349/224	3365/335	3324/223	3273/72
VE (95% CI)	74.7 (64.3–82.0)	54.5 (40.6–65.1)	60.9 (47.0–71.2)	70.3 (55.4–80.3)	62.0 (39.7–76.1)	49.7 (26.3–65.6)	61.1 (45.2–72.3)	59.6 (39.4–73.1)	67.1 (34.0–83.6)

*n*_u_, number of unvaccinated individuals; *n*_v_, number of vaccinated individuals whose time since last dose fell within the specific interval; VE, vaccine effectiveness; CI, confidence interval.

**Table 3.** Vaccine effectiveness against COVID-19 outcomes over different time intervals after third-dose COVID-19 vaccination.

Days since 3rd dose	0–13	14–30	31–60	61–90	91–120	121–150	151–180
COVID-19-related hospitalization							
**BNT162b2**							
Case (*n*_u_/*n*_v_)	18404/155	18310/246	18397/364	17987/266	17816/186	17615/223	17492/119
Control (*n*_u_/*n*_v_)	57041/3681	57344/3035	57689/3197	57217/1635	56816/913	56802/765	56378/372
VE (95% CI)	91.2 (89.5–92.6)	83.9 (81.4–86.1)	78.4 (75.7–80.9)	71.3 (66.7–75.2)	67.1 (60.4–72.6)	44.8 (33.6–54.1)	44.0 (26.9–57.1)
**CoronaVac**							
Case (*n*_u_/*n*_v_)	18404/354	18310/494	18397/673	17987/523	17816/331	17615/320	17492/133
Control (*n*_u_/*n*_v_)	57041/3585	57344/3100	57689/3328	57217/2026	56816/1343	56802/722	56378/283
VE (95% CI)	76.7 (73.7–79.4)	64.4 (60.4–68.1)	59.7 (55.7–63.4)	47.9 (41.8–53.4)	51.3 (44.2–57.5)	2.6 (−14.1 to 16.9)	10.2 (−15.6 to 30.3)
COVID-19-related mortality							
**BNT162b2**							
Case (*n*_u_/*n*_v_)	5786/5	5780/4	5767/7	5708/9	5674/2	5643/3	5621/2
Control (*n*_u_/*n*_v_)	20738/763	20795/609	20780/498	20761/241	20730/140	20731/36	20708/11
VE (95% CI)	98.2 (95.0–99.3)	97.4 (93.0–99.0)	95.7 (90.2–98.1)	90.9 (80.8–95.7)	94.6 (77.7–98.7)	73.7 (7.5–92.5)	56.5 (−122.6 to 91.5)
**CoronaVac**							
Case (*n*_u_/*n*_v_)	5786/8	5780/29	5767/28	5708/17	5674/11	5643/8	5621/1
Control (*n*_u_/*n*_v_)	20738/925	20795/721	20780/690	20761/315	20730/253	20731/95	20708/15
VE (95% CI)	96.7 (93.2–98.4)	87.6 (81.3–91.7)	88.6 (82.7–92.5)	82.9 (70.6–90.1)	86.4 (73.3–93.1)	76.5 (48.4–89.3)	82.0 (−47.0 to 97.8)
COVID-19-related severe complications							
**BNT162b2**							
Case (*n*_u_/*n*_v_)	1056/8	1057/10	1064/14	1024/10	1013/6	1002/7	997/3
Control (*n*_u_/*n*_v_)	3252/242	3272/193	3272/196	3278/95	3262/41	3244/32	3242/17
VE (95% CI)	93.1 (84.9–96.8)	91.0 (81.7–95.6)	89.3 (79.5–94.4)	80.0 (58.8–90.3)	75.7 (35.5–90.8)	62.5 (1.6–85.7)	69.0 (−13.2 to 91.5)
**CoronaVac**							
Case (*n*_u_/*n*_v_)	1056/10	1057/21	1064/25	1024/17	1013/11	1002/7	997/5
Control (*n*_u_/*n*_v_)	3252/228	3272/181	3272/198	3278/123	3262/77	3244/27	3242/9
VE (95% CI)	91.6 (83.3–95.8)	78.5 (63.7–87.3)	80.1 (66.6–88.1)	71.4 (49.0–83.9)	63.6 (29.4–81.2)	53.9 (−22.2 to 82.6)	−20.2 (−327.9 to 66.3)

*n*_u_, number of unvaccinated individuals; *n*_v_, number of vaccinated individuals whose time since last dose fell within the specific interval; VE, vaccine effectiveness; CI, confidence interval.

Similar trends were observed for VE after the third vaccine dose (Figure 2, Table 3). VE against COVID-19-related hospitalization decreased from 91.2% (89.5–92.6%) for BNT162b2 and 76.7% (73.7–79.4%) for CoronaVac at 0–13 days, to 67.1% (60.4–72.6%) for BNT162b2 and 51.3% (44.2–57.5%) for CoronaVac at 91–120 days after the third dose. For COVID-19-related mortality, VE for BNT162b2 remained consistently high from 0–13 days [98.2% (95.0–99.3%)] to 91–120 days [94.6% (77.7–98.7%)] after the third dose, whereas VE for CoronaVac reduced gradually from 0–13 days [96.7% (93.2–98.4%)] to 91–120 days [86.4% (73.3–93.1%)] after the third dose. VE against COVID-19-related severe complications was 93.1% (84.9–96.8%) for BNT162b2 and 91.6% (83.3–95.8%) for CoronaVac at 0–13 days; and reduced to 75.7% (35.5–90.8%) for BNT162b2 and 63.6% (29.4–81.2%) for CoronaVac at 91–120 days after the third dose. We found no significant risk reduction against COVID-19-related mortality and severe complications for both CoronaVac and BNT162b2 by 151–180 days after the third dose compared to the unvaccinated, albeit this should be interpreted with caution due to the limited number of events.

In general, consistent trends of waning VE were also observed in all subgroups (Supplementary Table 2). Notably, VE against COVID-19-related hospitalization was generally higher with slower waning among individuals aged ≥65 years who received two doses of BNT162b2. Results from the main analyses were robust to sensitivity analyses (Supplementary Table 3). Estimated linear trend in waning VE was consistent with the main findings (Supplementary Figure 2), but should not be interpreted as a formal comparison between different vaccine platforms or second versus third dose.

## Discussion

This study revealed that VE against severe and fatal COVID-19 with two to three doses of BNT162b2 and CoronaVac both decreased with time. The decline in VE was most notable within the first 90 days after the second dose, which is in line with the observation of waning immunity against Omicron infection after vaccination in other studies [26,27]. Although it was believed that protection against severe outcomes, in comparison to infection, generally persisted with time [4,5]. Our findings demonstrated a notable decline in VE after the third dose in terms of COVID-19-related hospitalization. Our findings highlight the importance of timely administration of additional doses in order to maintain protection against severe COVID-19.

In contrast to the previously reported effectiveness of ≥90% against COVID-19-related hospitalization and deaths after 20 weeks in the UK [5] and up to 6 months after the second dose in the US [28] against the Delta variant, our study recorded much lower values. During the current Omicron BA.2 epidemic in Hong Kong, the risk reduction with BNT162b2 observed at six months after the second dose was 61% against COVID-related hospitalization, 87% against COVID-19 mortality, and 85% against severe COVID-19 disease, respectively. This corresponds to the previous studies that described a lower VE against the Omicron variant when compared to earlier variants [29,30]. Our data are comparable with the observed effectiveness of BNT162b2 against hospitalization during the Omicron outbreak at 6–8 months in the US (42%, [34–50]) [29] and at 5–6 months in South Africa (46% [39–51]) [7]. VE of the third dose against hospitalization in our study was 67% (60–73) at 91–120 days and 45% (34–54) at 121–150 days, which is also similar to that in the US (4-<6 months: 66% [63–70]) and South Africa (3–4 months: 50% [4–74]) [7]. Meanwhile, the waning effectiveness of CoronaVac has been less discussed. An increase in the cumulative incidence of COVID-19 infection among CoronaVac recipients over time has been documented [31]. Another study conducted before the Omicron era compared COVID-19-related ICU admission and death rates between “early vaccinees” and “late vaccinees” and concluded that VE of CoronaVac against infection waned with time but effectiveness against mortality was persistent [32]. At present, direct evidence is lacking. Our study bridged the research gap by demonstrating a decline in effectiveness of CoronaVac during the Omicron outbreak with time. At six months after the second dose, the effectiveness of CoronaVac was 43% against COVID-19-related hospitalization, 69% against COVID-19 mortality, and 60% against severe complications, respectively. Waning VE was also observed among three-dose recipients, especially in COVID-19-related hospitalization and severe COVID-19, while effectiveness against COVID-19-related mortality remained high within 120 days after the third dose, reaching 95% and 86% in BNT162b2 and CoronaVac recipients, respectively. Owing to the small number of outcome events recorded, the estimated VE beyond 120 days after the third dose should be interpreted with caution.

In general, the trend of waning protection against severe COVID-19 corresponds to the decline in the titre of neutralizing antibody against Omicron and spike-specific CD4+ and CD8+ T cells in the serum of healthy volunteers three months after the second dose of vaccine [33]. It was observed that the titre of neutralizing antibody against Omicron decreased to the detection limit after three months among people who received CoronaVac [33]. While other studies reported that spike-specific antibodies could last more than 6 months after natural infection despite a rigorous decline after 6 weeks [34]. Nonetheless, some argued that the decline in humoral immunity does not necessarily predict a wane in vaccine protection [35]. Yet the exact mechanism remains to be elucidated. On the other hand, it was postulated that SARS-CoV-2-specific humoral immunity provides more persistent protection against severe COVID-19 [34,36]. In the present study, we demonstrated that while vaccine protection against severe COVID-19 continued to wane over 8 months after the second dose, a substantial degree of protection remains. Nevertheless, vaccination with the third dose of vaccine would be warranted to provide a higher level of protection.

Our findings demonstrated that VE of both BNT162b2 and CoronaVac waned over time. In particular, a greater extent of decline was noted in people who received CoronaVac. Considering the effectiveness against COVID-19-related mortality, both BNT162b2 and CoronaVac offered at least 90% protection at the beginning. However, the effectiveness of CoronaVac decreased to 59% after 3 months since the second dose of vaccination while that of BNT162b2 was maintained at 93%. Although inactivated vaccines were also shown to elicit T cell response in addition to humoral response [37], the disparity in the extent of T cell response has been reported in a study comparing blood samples from BNT162b2 recipients and CoronaVac recipients, which showed that more BNT162b2 recipients developed spike-specific CD4+ T cells and CD8+ T cells [33]. While some studies hypothesized that there might be qualitative differences in addition to the quantitative differences amongst the T cell response triggered by mRNA vaccine and inactivated vaccine [38], evidence that directly compares T cell response induced by BNT162b2 and CoronaVac remains limited, and further studies are warranted. In contrast, the effectiveness against mortality at 3 months after the third dose of BNT162b2 (94.6%) and CoronaVac (86.4%) was comparable, suggesting the importance of getting booster shots, especially in people who completed a primary series of CoronaVac.

By and large, waning immunity against Omicron after vaccination was observed. Nevertheless, booster shots were still largely effective. Based on a phase 4 randomized trial in Brazil, booster shots with either BNT162b2 or CoronaVac were able to raise IgG antibody levels substantially and increase neutralizing capacity against Omicron [26]. In the present study, the effectiveness of BNT162b2 against hospitalization was 33.9% at 8 months after the second dose, but the effectiveness was as high as 83.9% one month after the third dose. The effectiveness study in the US also suggested a rise in the effectiveness of BNT162b2 against hospitalization from 45% at 8 months after the third dose, to 76% shortly after the fourth dose among adults above 65 years of age [29]. This reinforces the importance of booster doses in combating different variants of concern during the COVID-19 pandemic.

This study is among the first to evaluate waning in VE of two to three doses of CoronaVac against Omicron in a Chinese population, where current evidence remains scarce in contrast to mRNA vaccines which are being studied more frequently. Owing to the suboptimal vaccine coverage in HK, this study enrolled a significant proportion of unvaccinated persons, hence allowing evaluation of the real-world protection of these vaccines. Our findings demonstrated that despite vaccine protection against severe or fatal COVID-19 waning significantly 8–9 months after the second dose, a substantial degree of protection remains. Nevertheless, timely vaccination with the third dose of vaccine, especially in those who received two doses of CoronaVac, would be warranted to provide a higher level of protection against severe COVID-19. Our results also shed some light on discussion of the optimal timing of the fourth vaccine dose.

This study has several limitations. First, VE beyond 180 days after the third dose of vaccine could not be estimated due to insufficient samples as it has been less than 10 months since the rollout of booster vaccination in the general population locally. Second, it was possible that some asymptomatic COVID-19 infections were not captured, since universal COVID-19 screening was not implemented in HK, as with most countries worldwide. Misclassification due to false negatives in PCR tests was also possible. However, PCR remains the gold standard for diagnosis owing to its high specificity >99% [24], and the risk of false negatives was minimal in the analysis for severe or fatal COVID-19 disease. Third, there might be underdiagnosis as the need for ventilatory support was determined by the procedural codes in the electronic database. The rate of ICU admission was limited by its maximal capacity, and we could not eliminate the possibility that some severe cases who deteriorated rapidly died before being transferred to ICU. Nonetheless, these cases would have been captured by the COVID-19-related death outcome. Fourth, this study did not account for possible differences in health-seeking behaviour among vaccinated and unvaccinated individuals that may potentially put them at a higher risk of contracting COVID-19. Further, as with any observational studies, the possibility of confounding and selection bias could not be ruled out. Lastly, it should be noted that the findings of this study may not be generalizable to other COVID-19 vaccines.

## Conclusion

Both CoronaVac and BNT162b2 were associated with a significant risk reduction against COVID-19-related hospitalization, death, and severe complications for at least 8 months after the second dose and 4 months after the third dose, when compared to the unvaccinated. However, a significant waning in VE over time was observed for both vaccines against COVID-19-related hospitalization and for CoronaVac against COVID-19-related mortality. Timely administration of booster doses could provide a higher level of protection against COVID-19-related hospitalization and mortality.

## Supplementary Material

Supplemental Material

## Data sharing

Data are not available as the data custodians (the Hospital Authority and the Department of Health of Hong Kong SAR) have not given permission for sharing due to patient confidentiality and privacy concerns. Local academic institutions, government departments, or non-governmental organizations may apply for the access to data through the Hospital Authority’s data sharing portal (https://www3.ha.org.hk/data).
