# Supplementary material for: Waning effectiveness against COVID-19-related hospitalization, severe complications, and mortality with two to three doses of CoronaVac and BNT162b2: a case–control study
Source: Emerg Microbes Infect. 2023 May 18;12(1):2209201. doi: 10.1080/22221751.2023.2209201 (PMC10197998; doi:10.1080/22221751.2023.2209201)
Supplement: Supplemental Material [file TEMI_A_2209201_SM2696.docx]

**Supplementary Appendix**

**Supplementary Table 1.** Vaccination programme priority groups rollout schedule - Hong Kong

| Order of expansion | Date of rollout | Vaccination group |
| --- | --- | --- |
| First^1^ | 26 Feb 2021 | - Healthcare workers and staff involved in anti-epidemic work - Persons aged 60 or above and a maximum of 2 carers accompanying older adults aged above 70 - Residents and staff of residential care homes for the elderly and persons with disabilities - People providing essential public services - People providing cross-boundary transportation or working at control points and ports |
| Second^2^ | 8 Mar 2021 | - Staff of food and beverages premises, markets, supermarkets, convenience stores, couriers, and takeaway delivery - Staff of local public transport operators - Registered construction workers - Property management staff - Teachers and school staff - Staff in the tourism industry - Staff of scheduled premises under the Prevention and Control of Disease |
| Third^3^ | 16 Mar 2021 | - People aged between 30 and 59 years - Students aged 16 years or above studying outside Hong Kong - Domestic helpers |
| Fourth^4^ | 15 Apr 2021 | - People aged ≥16 years eligible to receive BNT162b2 - People aged ≥18 years eligible to receive CoronaVac |
| Fifth^5^ | 10 Jun 2021 | - People aged ≥12 eligible to receive BNT162b2 |
| Sixth^6^ | 11 Nov 2021 | - Eligible persons under certain groups can receive a third dose of COVID-19 vaccine free of charge |
| Seventh^7^ | 20 Nov 2021 | - People aged 12 to 17 years eligible to receive CoronaVac |
| Eighth^8^ | 23 Nov 2021 | - Members of the public who have received two doses of the CoronaVac vaccine with the second dose received 6 months previously can make reservations for a third dose of a COVID-19 vaccine irrespective of certain groups |
| Ninth^9^ | 21 Jan 2022 | - People aged 5 to 11 years eligible to receive CoronaVac |
| Tenth^9^ | 16 Feb 2022 | - People aged 5 to 11 years eligible receive BNT162b2 |
| Eleventh^10^ | 15 Feb 2022 | - People aged 3 to 4 years eligible to receive CoronaVac |
| Twelfth^11^ | 05 Mar 2022 | - People aged ≥60 years eligible to receive a third dose at 3 months (reduced from 6 months) after the second dose - People aged 5 to 17 years eligible to receive a second dose of BNT162b2 at 8 weeks (reduced from 12 weeks) after the first dose - Immunocompromised people aged ≤12 years can make reservations for a third dose of a COVID-19 vaccine 4 weeks after receiving the first two doses |
| Thirteenth^9,10^ | 11 Mar 2022 | - People who received two doses of CoronaVac eligible to receive a third dose of CoronaVac or BioNTech vaccine 3 months after the second dose - People who received two doses of BioNTech eligible to receive a third dose of BioNTech or CoronaVac vaccine 5 months after the second dose - People aged 12 to 17 years who have received two doses of BioNTech vaccine eligible to receive a third dose of BioNTech or CoronaVac vaccine 5 months after the second dose |
| Fourteenth^9^ | 21 Mar 2022 | - Immunocompromised people aged ≥12 years who have received three doses of COVID-19 vaccine may receive a fourth vaccine dose at least 3 months after their last dose |
| Fifthteenth^11^ | 14 Apr 2022 | - Children aged 3 to 11 years who have received two doses of CoronaVac eligible to receive a third dose after 3 months - Persons aged 60 years or above who have received three doses of the CoronaVac or BioNTech vaccine eligible to receive a fourth dose at least 3 months after the last dose |
| Sixteenth^12^ | 21 May 2022 | - Uninfected individuals aged 18 to 59 years who are at higher risk of COVID-19 exposure or have personal needs may choose to receive a fourth dose of COVID-19 vaccine, regardless of whether they have received BioNTech or CoronaVac vaccine as their previous doses |
| Seventeenth^13^ | 4 Aug 2022 | - Children aged 6 months to 3 years eligible to receive the CoronaVac vaccine - Persons aged 50 to 59 years who have received three doses of CoronaVac or BioNTech vaccine eligible to receive a fourth dose at least 3 months after the last dose |

**References:**

1. HKSAR. Government announces 2019 COVID-19 Vaccination Programme. Press Releases. 18 Feb 2021 (https://www.info.gov.hk/gia/general/202102/18/P2021021800767.htm?fontSize=1)

2. HKSAR. Government expands scope of priority groups and opens more CVCs. Press Releases. 8 Mar 2021 (https://www.info.gov.hk/gia/general/202103/08/P2021030800738.htm)

3. HKSAR. Vaccination priority groups to be expanded to cover people aged 30 or above. Press Releases. 15 Mar 2021 (https://www.info.gov.hk/gia/general/202103/15/P2021031500626.htm?fontSize=1)

4. HKSAR. COVID-19 Vaccination Programme opens to persons aged 16 or above. Press Releases. 15 Apr 2021 (https://www.info.gov.hk/gia/general/202104/15/P2021041500565.htm?fontSize=1)

5. HKSAR. Persons aged 12 to 15 can make reservations to receive BioNTech vaccine from tomorrow. Press Releases. 10 Jun 2021 (https://www.info.gov.hk/gia/general/202106/10/P2021061000556.htm?fontSize=1)

6. Third dose COVID-19 vaccination arrangements for persons under certain groups. Press Releases. 3 Nov 2021 (https://www.info.gov.hk/gia/general/202111/03/P2021110300536.htm)

7. SFH approves lowering age limit for receiving CoronaVac vaccine. Press Releases. 20 Nov 2021 (https://www.info.gov.hk/gia/general/202111/20/P2021112000292.htm)

8. Government extends third dose COVID-19 vaccination arrangements. Press Releases. 18 Nov 2021 (https://www.info.gov.hk/gia/general/202111/18/P2021111800310.htm)

9. HKSAR. Arrangements for children aged 5 to 11 to receive COVID-19 vaccines. Press Releases. 20 Jan 2022 (https://www.info.gov.hk/gia/general/202201/20/P2022012000714.htm)

10. HKSAR. Lowering of minimum age for receiving Sinovac vaccine to three years old starting from February 15. Press Releases. 13 Feb 2022 (<https://www.info.gov.hk/gia/general/202202/13/P2022021300644.htm>)

11. HKSAR. Third dose Sinovac vaccine booking arrangements for children aged 3 to 11. Press Releases. 13 Apr 2022 (<https://www.info.gov.hk/gia/general/202204/13/P2022041300609.htm>)

12. HKSAR. Persons aged 18 to 59 may choose to receive fourth dose of COVID-19 vaccine. Press Releases. 21 May 2022 (<https://www.info.gov.hk/gia/general/202205/21/P2022052000831.htm>)

13. HKSAR. COVID-19 vaccination arrangements for children aged six months or above and for persons aged from 50 to 59 receiving fourth dose Press Releases. 2 Aug 2022 (https://www.info.gov.hk/gia/general/202208/02/P2022080200699.htm)

**Supplementary Table 2. Subgroup analyses**

1. **After vaccination with two doses**

|  | Vaccine effectiveness (%) (95% CI) | | | | | | | | |
| --- | --- | --- | --- | --- | --- | --- | --- | --- | --- |
|  | 0-13 days | 14-30 days | 31-60 days | 61-90 days | 91-120 days | 121-150 days | 151-180 days | 181-210 days | 211-240 days |
| *COVID-19 related hospitalisation* | | | | | | | | | |
| **BNT162b2** | | | | | | | | | |
| Age < 65 | 75.0 (66.4-81.3) | 69.5 (61.1-76.1) | 56.9 (47.7-64.5) | 59.7 (49.5-67.9) | 57.7 (47.0-66.3) | 36.0 (26.6-44.3) | 53.7 (48.5-58.4) | 50.9 (45.6-55.6) | 37.7 (29.8-44.7) |
| Age ≥ 65 | 83.7 (79.9-86.8) | 74.5 (70.1-78.3) | 78.1 (74.7-81.1) | 74.5 (69.7-78.5) | 60.2 (53.0-66.3) | 62.6 (55.6-68.4) | 77.5 (73.7-80.8) | 75.8 (71.3-79.6) | 65.2 (56.0-72.4) |
| Male | 82.5 (77.9-86.2) | 74.5 (69.4-78.7) | 73.6 (69.1-77.4) | 69.1 (63.0-74.2) | 63.2 (55.7-69.5) | 54.8 (47.4-61.1) | 65.2 (60.6-69.3) | 60.1 (54.7-64.7) | 43.0 (33.9-50.9) |
| Female | 79.4 (73.8-83.8) | 70.8 (64.5-76.0) | 71.6 (66.5-75.9) | 71.9 (65.6-77.1) | 53.3 (43.6-61.4) | 44.2 (35.4-51.8) | 64.2 (59.7-68.2) | 61.5 (56.8-65.7) | 49.3 (41.4-56.1) |
| CCI < 2 | 83.4 (79.0-86.8) | 75.0 (70.1-79.0) | 75.9 (71.8-79.4) | 70.7 (64.9-75.5) | 59.0 (51.6-65.4) | 45.8 (38.9-51.9) | 60.3 (56.3-64.0) | 57.4 (53.2-61.2) | 46.0 (39.5-51.8) |
| CCI ≥ 2 | 76.6 (68.8-82.5) | 65.4 (56.6-72.4) | 63.9 (56.3-70.2) | 68.1 (59.4-74.9) | 65.0 (54.2-73.3) | 61.0 (49.0-70.1) | 75.3 (68.4-80.7) | 72.0 (63.5-78.4) | 37.7 (14.8-54.5) |
| **CoronaVac** | | | | | | | | | |
| Age < 65 | 75.1 (67.8-80.8) | 62.5 (53.5-69.7) | 55.9 (47.1-63.2) | 51.2 (39.2-60.8) | 44.0 (30.2-55.1) | 30.6 (18.4-40.9) | 41.8 (34.0-48.7) | 29.0 (19.9-37.2) | 25.0 (12.3-35.9) |
| Age ≥ 65 | 71.5 (69.1-73.8) | 66.4 (63.8-68.8) | 63.1 (60.3-65.7) | 50.8 (46.1-55.0) | 23.6 (16.0-30.6) | 33.0 (26.2-39.1) | 50.1 (45.3-54.5) | 50.3 (44.1-55.8) | 45.8 (34.3-55.3) |
| Male | 73.1 (70.0-75.9) | 66.7 (63.2-69.9) | 62.7 (58.9-66.1) | 56.9 (51.3-61.9) | 27.2 (17.8-35.5) | 37.2 (29.6-44.0) | 54.9 (49.9-59.4) | 46.9 (40.4-52.7) | 37.5 (26.3-46.9) |
| Female | 70.7 (67.2-73.8) | 65.2 (61.5-68.5) | 61.7 (57.9-65.2) | 44.3 (37.7-50.3) | 28.7 (19.0-37.3) | 26.8 (17.6-35.0) | 39.8 (33.2-45.7) | 36.8 (28.7-44.0) | 34.3 (21.7-44.9) |
| CCI < 2 | 70.3 (67.1-73.3) | 64.8 (61.4-67.9) | 60.2 (56.5-63.6) | 46.8 (40.7-52.2) | 23.0 (13.8-31.1) | 22.0 (13.6-29.5) | 38.7 (33.0-43.9) | 38.8 (32.4-44.5) | 35.7 (25.9-44.2) |
| CCI ≥ 2 | 71.9 (67.6-75.6) | 65.8 (61.2-69.8) | 63.6 (58.7-67.9) | 52.9 (44.9-59.7) | 39.4 (27.3-49.4) | 49.6 (39.4-58.0) | 61.6 (54.5-67.7) | 39.2 (26.2-49.9) | 32.4 (9.0-49.7) |
| *COVID-19 related mortality* | | | | | | | | | |
| **BNT162b2** | | | | | | | | | |
| Age < 65 | 87.7 (45.9-97.2) | 96.1 (61.5-99.6) | 71.3 (21.1-89.6) | 95.4 (67.1-99.4) | 93.1 (55.4-98.9) | 95.0 (75.5-99.0) | 84.1 (61.9-93.4) | 86.7 (72.3-93.6) | 59.1 (11.0-81.2) |
| Age ≥ 65 | 95.4 (90.5-97.8) | 89.6 (83.2-93.6) | 89.2 (83.7-92.9) | 92.6 (86.7-95.9) | 91.8 (83.0-96.0) | 83.0 (70.2-90.3) | 88.7 (81.2-93.2) | 88.3 (79.3-93.4) | 82.0 (61.9-91.5) |
| Male | 96.2 (90.5-98.5) | 89.2 (80.2-94.1) | 86.2 (78.6-91.2) | 91.5 (83.9-95.6) | 92.6 (84.0-96.6) | 87.5 (75.8-93.6) | 89.7 (81.8-94.2) | 86.7 (77.3-92.2) | 72.1 (50.2-84.3) |
| Female | 91.2 (78.2-96.4) | 90.3 (79.6-95.4) | 91.5 (82.0-96.0) | 95.6 (85.7-98.6) | 93.0 (70.6-98.4) | 83.3 (60.1-93.0) | 83.2 (66.9-91.4) | 88.7 (75.3-94.9) | 81.2 (36.1-94.5) |
| CCI < 2 | 94.7 (83.1-98.3) | 91.8 (83.6-95.9) | 90.8 (81.7-95.4) | 96.5 (88.5-99.0) | 93.9 (82.7-97.9) | 87.3 (71.5-94.3) | 95.8 (89.3-98.4) | 90.9 (81.6-95.6) | 82.2 (58.6-92.4) |
| CCI ≥ 2 | 94.1 (86.3-97.5) | 88.6 (76.0-94.6) | 83.2 (72.3-89.8) | 87.3 (75.7-93.3) | 95.3 (83.5-98.7) | 86.3 (67.6-94.2) | 70.0 (49.0-82.3) | 78.9 (61.1-88.6) | 45.7 (-14.0-74.1) |
| **CoronaVac** | | | | | | | | | |
| Age < 65 | 97.1 (78.6-99.6) | 92.9 (73.4-98.1) | 84.9 (62.9-93.9) | 66.7 (-0.9-89.0) | 92.3 (37.3-99.1) | 74.2 (23.7-91.3) | 52.6 (10.8-74.8) | 82.2 (60.4-92.0) | 86.0 (48.9-96.2) |
| Age ≥ 65 | 89.8 (86.9-92.1) | 80.5 (76.6-83.7) | 73.2 (68.3-77.4) | 63.9 (55.1-71.0) | 56.7 (43.1-67.1) | 55.7 (43.5-65.3) | 58.1 (48.2-66.1) | 66.6 (54.6-75.4) | 74.6 (54.9-85.6) |
| Male | 90.8 (87.0-93.4) | 82.0 (77.1-85.8) | 70.7 (63.6-76.3) | 66.4 (55.1-74.9) | 60.1 (42.8-72.1) | 59.5 (45.3-70.0) | 67.1 (56.8-74.9) | 69.7 (57.1-78.6) | 79.5 (62.0-89.0) |
| Female | 89.5 (84.7-92.8) | 79.7 (73.3-84.6) | 77.2 (70.4-82.4) | 61.1 (46.7-71.6) | 57.9 (36.4-72.1) | 53.4 (31.0-68.5) | 39.5 (18.2-55.2) | 68.5 (47.9-81.0) | 65.3 (12.7-86.2) |
| CCI < 2 | 88.0 (83.1-91.4) | 81.5 (75.7-85.9) | 70.5 (62.5-76.8) | 63.7 (49.9-73.8) | 57.1 (35.8-71.4) | 69.3 (55.6-78.8) | 52.0 (36.3-63.8) | 78.0 (65.6-86.0) | 80.2 (53.4-91.6) |
| CCI ≥ 2 | 93.6 (89.8-95.9) | 81.0 (74.8-85.7) | 78.4 (71.5-83.7) | 64.5 (50.8-74.4) | 60.5 (38.4-74.7) | 53.1 (31.7-67.8) | 59.8 (43.0-71.6) | 52.1 (25.5-69.2) | 65.8 (29.8-83.4) |
| *COVID-19 related severe complications* | | | | | | | | | |
| **BNT162b2** | | | | | | | | | |
| Age < 65 | 83.6 (27.3-96.3) | 73.1 (3.7-92.5) | 29.5 (-70.7-70.9) | 85.6 (48.3-96.0) | 66.3 (-11.6-89.8) | 83.5 (47.1-94.9) | 91.2 (76.2-96.7) | 91.6 (78.6-96.7) | 68.2 (33.0-84.9) |
| Age ≥ 65 | 80.8 (53.9-92.0) | 69.6 (44.9-83.2) | 76.9 (53.2-88.6) | 71.9 (40.8-86.6) | 80.0 (43.1-93.0) | 59.8 (8.2-82.4) | 79.4 (58.0-89.9) | 79.3 (52.6-91.0) | 81.9 (25.6-95.6) |
| Male | 79.3 (55.6-90.4) | 70.5 (45.7-83.9) | 74.4 (47.1-87.7) | 73.0 (42.2-87.4) | 91.7 (64.9-98.0) | 78.8 (48.1-91.3) | 83.0 (66.1-91.5) | 81.1 (61.6-90.7) | 76.6 (42.8-90.4) |
| Female | 91.4 (36.0-98.8) | 68.1 (22.8-86.8) | 63.4 (26.5-81.8) | 82.6 (53.9-93.5) | 54.4 (-17.5-82.3) | 65.2 (13.2-86.0) | 88.6 (72.3-95.3) | 91.0 (73.2-97.0) | 66.2 (16.3-86.3) |
| CCI < 2 | 79.0 (51.6-90.9) | 74.0 (50.5-86.3) | 67.2 (37.6-82.8) | 87.2 (66.0-95.2) | 74.2 (37.8-89.3) | 65.9 (29.6-83.5) | 88.2 (75.9-94.2) | 86.9 (73.2-93.5) | 65.0 (30.9-82.3) |
| CCI ≥ 2 | 86.4 (41.3-96.9) | 67.0 (13.8-87.3) | 45.0 (-29.5-76.6) | 44.3 (-42.2-78.2) | 64.7 (-62.2-92.3) | 83.4 (18.8-96.6) | 69.8 (1.1-90.8) | 68.0 (4.5-89.3) | 79.2 (-73.0-97.5) |
| **CoronaVac** | | | | | | | | | |
| Age < 65 | 82.6 (47.5-94.2) | 56.7 (-9.0-82.8) | 44.1 (-29.5-75.8) | 64.7 (1.4-87.4) | 85.8 (35.5-96.9) | 71.3 (20.5-89.6) | 39.2 (-12.0-67.0) | 86.9 (66.6-94.8) | 68.1 (11.3-88.6) |
| Age ≥ 65 | 71.3 (57.8-80.5) | 52.5 (35.6-64.9) | 60.2 (43.2-72.1) | 71.3 (53.7-82.2) | 51.7 (18.4-71.4) | 44.6 (13.7-64.5) | 65.9 (46.5-78.2) | 33.7 (-6.9-58.9) | 75.0 (25.3-91.6) |
| Male | 65.1 (47.4-76.8) | 52.1 (32.7-65.8) | 67.6 (51.4-78.4) | 71.6 (50.2-83.8) | 71.5 (45.5-85.0) | 51.3 (21.0-70.0) | 59.3 (37.6-73.5) | 43.0 (10.5-63.7) | 44.8 (-21.5-75.0) |
| Female | 86.4 (73.5-93.0) | 58.3 (35.5-73.0) | 49.7 (20.1-68.4) | 69.7 (45.1-83.3) | 45.1 (-8.7-72.3) | 50.0 (6.4-73.3) | 65.3 (38.4-80.4) | 86.2 (62.0-95.0) | 90.2 (54.5-97.9) |
| CCI < 2 | 76.5 (62.6-85.3) | 54.6 (36.0-67.8) | 64.4 (46.3-76.4) | 83.2 (67.5-91.3) | 63.4 (32.4-80.1) | 28.0 (-12.2-53.8) | 56.9 (35.8-71.1) | 60.8 (36.5-75.8) | 77.2 (43.0-90.9) |
| CCI ≥ 2 | 66.6 (39.7-81.5) | 46.2 (12.9-66.8) | 58.5 (28.7-75.9) | 58.8 (17.1-79.5) | 70.9 (23.7-88.9) | 73.8 (34.0-89.6) | 74.9 (37.8-89.9) | 39.2 (-51.1-75.6) | -34.2 (-393.3-63.5) |

1. **After vaccination with three doses**

|  | Vaccine effectiveness (%) (95% CI) | | | | | | |
| --- | --- | --- | --- | --- | --- | --- | --- |
|  | 0-13 days | 14-30 days | 31-60 days | 61-90 days | 91-120 days | 121-150 days | 151-180 days |
| *COVID-19 related hospitalisation* | | | | | | | |
| **BNT162b2** | | | | | | | |
| Age < 65 | 88.3 (85.2-90.7) | 78.5 (73.9-82.2) | 75.8 (71.4-79.5) | 71.4 (64.6-76.8) | 70.9 (61.6-78.0) | 58.1 (44.3-68.5) | 65.1 (48.7-76.3) |
| Age ≥ 65 | 93.6 (91.7-95.1) | 88.1 (85.2-90.5) | 81.3 (77.6-84.4) | 70.7 (63.9-76.2) | 64.0 (53.8-71.9) | 36.0 (18.1-50.0) | 13.6 (-25.4-40.5) |
| Male | 91.1 (88.8-92.9) | 83.0 (79.4-85.9) | 75.6 (71.4-79.1) | 68.7 (62.3-74.1) | 68.5 (59.7-75.4) | 38.6 (21.3-52.1) | 32.8 (2.9-53.5) |
| Female | 91.2 (88.7-93.2) | 85.0 (81.3-87.9) | 81.6 (77.7-84.7) | 74.8 (67.9-80.1) | 64.9 (53.8-73.3) | 51.4 (35.9-63.2) | 53.3 (31.3-68.3) |
| CCI < 2 | 90.8 (88.7-92.4) | 83.6 (80.6-86.1) | 76.4 (72.9-79.4) | 74.1 (69.2-78.3) | 70.0 (62.5-76.0) | 46.7 (34.5-56.7) | 47.2 (29.1-60.6) |
| CCI ≥ 2 | 89.8 (85.1-93.0) | 82.8 (75.7-87.8) | 80.0 (72.6-85.4) | 63.5 (48.1-74.4) | 50.5 (23.9-67.8) | 40.5 (-6.7-66.8) | -12.0 (-216.4-60.4) |
| **CoronaVac** | | | | | | | |
| Age < 65 | 78.8 (72.8-83.5) | 60.9 (52.1-68.0) | 65.0 (58.2-70.8) | 41.7 (29.8-51.5) | 54.8 (41.5-65.0) | 37.5 (15.4-53.8) | 42.4 (13.5-61.6) |
| Age ≥ 65 | 75.8 (72.3-78.9) | 65.2 (60.5-69.4) | 56.2 (50.8-60.9) | 51.0 (43.6-57.4) | 49.2 (40.3-56.8) | -16.1 (-40.0-3.7) | -19.3 (-65.2-13.9) |
| Male | 77.4 (73.4-80.7) | 68.2 (62.9-72.7) | 59.9 (54.4-64.8) | 47.2 (38.9-54.3) | 54.9 (46.3-62.2) | -3.8 (-27.7-15.7) | 8.8 (-28.8-35.4) |
| Female | 75.7 (70.9-79.7) | 60.0 (53.4-65.6) | 59.4 (53.0-64.9) | 49.0 (39.5-57.0) | 44.5 (31.0-55.3) | 10.0 (-15.2-29.7) | 11.1 (-28.8-38.7) |
| CCI < 2 | 73.4 (69.2-76.9) | 62.2 (56.9-66.8) | 58.8 (53.7-63.4) | 46.0 (38.5-52.6) | 53.6 (45.2-60.8) | 7.2 (-11.7-22.9) | 16.1 (-10.4-36.3) |
| CCI ≥ 2 | 80.9 (74.8-85.6) | 64.1 (54.3-71.9) | 57.2 (47.0-65.4) | 51.4 (35.2-63.6) | 43.8 (22.2-59.4) | -69.8 (-157.5--12.0) | 12.9 (-133.7-67.6) |
| *COVID-19 related mortality* | | | | | | | |
| **BNT162b2** | | | | | | | |
| Age < 65 | 97.8 (83.6-99.7) | - | 90.6 (72.0-96.8) | 94.5 (53.6-99.3) | - | 7.4 (-490.3-85.5) | 74.0 (-169.9-97.5) |
| Age ≥ 65 | 98.3 (94.6-99.5) | 96.6 (90.8-98.7) | 98.0 (91.9-99.5) | 89.1 (76.0-95.1) | 93.6 (73.4-98.5) | 90.6 (24.8-98.8) | 39.4 (-585.0-94.6) |
| Male | 98.1 (93.8-99.4) | 97.3 (91.5-99.1) | 95.2 (88.2-98.0) | 88.1 (72.9-94.8) | 91.4 (64.0-97.9) | 61.2 (-45.7-89.7) | 62.2 (-279.5-96.2) |
| Female | 98.4 (88.2-99.8) | 97.7 (83.1-99.7) | 97.1 (79.1-99.6) | 96.5 (73.8-99.5) | - | - | 48.1 (-436.4-95.0) |
| CCI < 2 | 98.1 (92.4-99.5) | 97.8 (90.9-99.5) | 97.4 (91.1-99.2) | 94.2 (81.0-98.2) | 95.9 (68.3-99.5) | 50.3 (-107.3-88.1) | 70.3 (-186.1-96.9) |
| CCI ≥ 2 | 97.2 (88.1-99.3) | 95.7 (82.4-98.9) | 94.5 (80.1-98.5) | 90.6 (63.8-97.5) | 90.5 (29.0-98.7) | - | 46.2 (-520.2-95.3) |
| **CoronaVac** | | | | | | | |
| Age < 65 | 96.8 (73.9-99.6) | 88.2 (64.8-96.0) | - | 75.9 (14.5-93.2) | - | 65.5 (-327.2-97.2) | - |
| Age ≥ 65 | 96.9 (93.3-98.5) | 87.6 (80.8-92.0) | 86.9 (80.0-91.5) | 83.2 (69.5-90.8) | 83.9 (68.0-91.9) | 77.3 (47.8-90.1) | 71.7 (-143.7-96.7) |
| Male | 96.3 (92.0-98.3) | 86.1 (77.5-91.4) | 90.5 (83.7-94.5) | 81.0 (63.7-90.0) | 86.6 (71.2-93.7) | 81.5 (46.3-93.6) | 79.9 (-69.0-97.6) |
| Female | 98.1 (86.5-99.7) | 90.8 (80.0-95.7) | 83.9 (68.1-91.9) | 86.6 (62.9-95.2) | 86.2 (40.2-96.8) | 66.6 (-13.4-90.2) | - |
| CCI < 2 | 98.1 (93.7-99.4) | 92.8 (85.6-96.4) | 90.8 (82.4-95.2) | 88.8 (74.8-95.0) | 93.7 (78.8-98.2) | 88.5 (47.1-97.5) | - |
| CCI ≥ 2 | 95.3 (86.9-98.3) | 75.3 (56.6-85.9) | 81.8 (67.0-89.9) | 63.5 (17.5-83.8) | 58.5 (-2.1-83.1) | 31.9 (-109.4-77.9) | - |
| *COVID-19 related severe complications* | | | | | | | |
| **BNT162b2** | | | | | | | |
| Age < 65 | 89.7 (70.3-96.4) | 88.7 (68.0-96.0) | 89.9 (73.1-96.2) | 95.9 (67.0-99.5) | 92.6 (35.4-99.2) | 80.3 (-85.4-97.9) | 51.6 (-158.8-91.0) |
| Age ≥ 65 | 96.5 (85.4-99.1) | 94.5 (82.1-98.3) | 88.9 (72.7-95.5) | 71.8 (36.9-87.4) | 60.7 (-19.9-87.1) | 54.3 (-39.7-85.1) | 76.6 (-115.8-97.5) |
| Male | 93.4 (83.5-97.4) | 93.0 (80.2-97.5) | 87.5 (71.8-94.5) | 66.8 (26.9-84.9) | 64.1 (-10.1-88.3) | 54.2 (-40.6-85.1) | 58.7 (-109.6-91.9) |
| Female | 92.4 (67.5-98.2) | 88.4 (68.0-95.8) | 91.4 (75.1-97.0) | 96.3 (70.1-99.5) | 90.0 (19.5-98.8) | 75.9 (-55.7-96.3) | 85.7 (-29.8-98.4) |
| CCI < 2 | 94.3 (84.6-97.9) | 90.0 (78.1-95.4) | 91.5 (80.5-96.3) | 76.1 (44.2-89.8) | 69.7 (15.9-89.1) | 60.4 (-13.8-86.2) | 78.2 (-5.2-95.5) |
| CCI ≥ 2 | 87.3 (46.5-97.0) | 92.5 (43.0-99.0) | 83.6 (34.7-95.9) | 75.5 (-21.4-95.0) | - | 42.1 (-888.7-96.6) | - |
| **CoronaVac** | | | | | | | |
| Age < 65 | 93.5 (75.2-98.3) | 85.5 (55.0-95.3) | 85.3 (58.6-94.8) | 67.1 (1.8-88.9) | 90.5 (21.5-98.8) | - | -72.5 (-1275.2-78.4) |
| Age ≥ 65 | 91.2 (80.1-96.1) | 74.4 (54.0-85.8) | 77.1 (58.4-87.3) | 72.9 (46.0-86.4) | 55.7 (10.5-78.1) | 37.0 (-72.4-77.0) | -3.6 (-425.1-79.5) |
| Male | 91.0 (79.9-95.9) | 75.5 (54.0-87.0) | 80.7 (61.4-90.4) | 66.5 (34.5-82.8) | 54.3 (6.7-77.6) | 53.2 (-50.7-85.4) | -293.4 (-2091.8-29.4) |
| Female | 94.0 (74.8-98.6) | 83.3 (57.5-93.4) | 80.0 (56.0-90.9) | 80.9 (37.0-94.2) | 87.1 (0.8-98.3) | 59.1 (-146.4-93.2) | - |
| CCI < 2 | 95.3 (87.2-98.2) | 78.6 (59.5-88.6) | 84.8 (70.2-92.3) | 63.9 (31.2-81.1) | 64.3 (21.0-83.8) | 26.8 (-111.8-74.7) | 39.0 (-194.2-87.3) |
| CCI ≥ 2 | - | 69.7 (6.0-90.2) | 57.6 (-17.3-84.7) | - | 65.4 (-64.9-92.7) | - | 38.3 (-710.1-95.3) |

CI: confidence interval; CCI: Charlson Comorbidity Index

**Supplementary Table 3**. Sensitivity analysis: inclusion of RAT-positive cases

1. **After vaccination with two doses**

| **Days since 2^nd^ dose** | **0-13** | **14-30** | **31-60** | **61-90** | **91-120** | **121-150** | **151-180** | **181-210** | **211-240** |
| --- | --- | --- | --- | --- | --- | --- | --- | --- | --- |
| *COVID-19 related hospitalisation* | | | | | | | | | |
| **BNT162b2** |  |  |  |  |  |  |  |  |  |
| Case (n_u_/n_v_) | 18524 / 161 | 18675 / 285 | 18795 / 417 | 18509 / 290 | 18363 / 321 | 18430 / 528 | 18787 / 781 | 18786 / 852 | 18417 / 613 |
| Control (n_u_/n_v_) | 59845 / 2394 | 60711 / 2915 | 61124 / 3787 | 59816 / 2444 | 59734 / 1896 | 60439 / 2757 | 61090 / 5666 | 60467 / 5211 | 59219 / 2447 |
| VE (95% CI) | 78.6 (74.4-82.1) | 68.9 (64.3-72.8) | 68.0 (64.1-71.5) | 69.5 (65.0-73.5) | 55.1 (48.6-60.8) | 44.4 (38.1-50.1) | 61.6 (58.1-64.9) | 56.6 (52.6-60.2) | 40.6 (34.1-46.5) |
| **CoronaVac** |  |  |  |  |  |  |  |  |  |
| Case (n_u_/n_v_) | 18524 / 808 | 18675 / 1116 | 18795 / 1317 | 18509 / 882 | 18363 / 842 | 18430 / 892 | 18787 / 1101 | 18786 / 885 | 18417 / 440 |
| Control (n_u_/n_v_) | 59845 / 9248 | 60711 / 10028 | 61124 / 9999 | 59816 / 4741 | 59734 / 3111 | 60439 / 3790 | 61090 / 5804 | 60467 / 3967 | 59219 / 1556 |
| VE (95% CI) | 69.2 (66.5-71.6) | 61.7 (58.7-64.4) | 60.0 (57.1-62.7) | 48.5 (43.7-52.8) | 26.5 (19.3-33.1) | 27.2 (20.6-33.2) | 45.5 (41.0-49.6) | 38.5 (32.9-43.7) | 30.5 (21.5-38.5) |
| *COVID-19 related mortality* | | | | | | | | | |
| **BNT162b2** |  |  |  |  |  |  |  |  |  |
| Case (n_u_/n_v_) | 5935 / 9 | 5953 / 19 | 5967 / 36 | 5901 / 14 | 5858 / 12 | 5867 / 16 | 5932 / 25 | 5916 / 33 | 5844 / 23 |
| Control (n_u_/n_v_) | 21390 / 638 | 21702 / 770 | 21822 / 1054 | 21597 / 568 | 21411 / 413 | 21515 / 432 | 21678 / 733 | 21408 / 621 | 21234 / 240 |
| VE (95% CI) | 95.3 (85.5-98.5) | 88.4 (73.0-95.0) | 83.4 (70.1-90.8) | 91.5 (78.9-96.6) | 87.6 (68.4-95.1) | 85.8 (67.2-93.8) | 91.7 (82.4-96.1) | 75.1 (55.6-86.1) | 72.0 (35.1-87.9) |
| **CoronaVac** |  |  |  |  |  |  |  |  |  |
| Case (n_u_/n_v_) | 5935 / 72 | 5953 / 170 | 5967 / 199 | 5901 / 137 | 5858 / 84 | 5867 / 108 | 5932 / 149 | 5916 / 75 | 5844 / 23 |
| Control (n_u_/n_v_) | 21390 / 2939 | 21702 / 3448 | 21822 / 3110 | 21597 / 1258 | 21411 / 727 | 21515 / 890 | 21678 / 1321 | 21408 / 810 | 21234 / 235 |
| VE (95% CI) | 84.7 (77.6-89.6) | 74.3 (65.8-80.7) | 65.3 (53.8-73.9) | 62.1 (45.4-73.7) | 59.3 (35.2-74.4) | 53.8 (31.6-68.8) | 54.9 (37.7-67.3) | 62.1 (41.5-75.4) | 67.8 (28.4-85.5) |
| *COVID-19 related severe complications* | | | | | | | | | |
| **BNT162b2** |  |  |  |  |  |  |  |  |  |
| Case (n_u_/n_v_) | 1058 / 10 | 1065 / 22 | 1058 / 22 | 1055 / 14 | 1045 / 9 | 1052 / 12 | 1069 / 16 | 1069 / 18 | 1040 / 13 |
| Control (n_u_/n_v_) | 3412 / 149 | 3536 / 187 | 3466 / 212 | 3375 / 135 | 3371 / 108 | 3404 / 120 | 3409 / 263 | 3381 / 231 | 3318 / 107 |
| VE (95% CI) | 78.2 (56.2-89.2) | 67.8 (45.1-81.1) | 67.7 (46.0-80.7) | 77.4 (56.0-88.4) | 70.3 (36.8-86.0) | 67.7 (39.0-82.9) | 84.4 (72.2-91.2) | 83.4 (70.2-90.7) | 80.3 (60.7-90.2) |
| **CoronaVac** |  |  |  |  |  |  |  |  |  |
| Case (n_u_/n_v_) | 1058 / 46 | 1065 / 79 | 1058 / 70 | 1055 / 35 | 1045 / 28 | 1052 / 43 | 1069 / 52 | 1069 / 38 | 1040 / 11 |
| Control (n_u_/n_v_) | 3412 / 521 | 3536 / 565 | 3466 / 514 | 3375 / 270 | 3371 / 185 | 3404 / 231 | 3409 / 339 | 3381 / 226 | 3318 / 71 |
| VE (95% CI) | 68.1 (54.7-77.6) | 50.5 (34.1-62.9) | 57.8 (41.6-69.6) | 67.6 (50.4-78.8) | 55.7 (28.8-72.5) | 45.9 (20.0-63.4) | 60.3 (43.1-72.4) | 54.3 (30.3-70.1) | 64.0 (23.7-83.1) |

n_u_: number of unvaccinated individuals; n_v_: number of vaccinated individuals whose time since last dose fell within the specific interval; VE: vaccine effectiveness; CI: confidence interval

1. **After vaccination with three doses**

| **Days since 3^rd^ dose** | **0-13** | **14-30** | **31-60** | **61-90** | **91-120** | **121-150** | **151-180** | |
| --- | --- | --- | --- | --- | --- | --- | --- | --- |
| *COVID-19 related hospitalisation* | | | | | | | |  |
| **BNT162b2** |  |  |  |  |  |  |  | |
| Case (n_u_/n_v_) | 18766 / 166 | 18702 / 261 | 18779 / 395 | 18417 / 292 | 18206 / 207 | 18003 / 243 | 17860 / 139 | |
| Control (n_u_/n_v_) | 58272 / 3774 | 58565 / 3139 | 58989 / 3295 | 58490 / 1761 | 58084 / 976 | 57977 / 831 | 57622 / 408 | |
| VE (95% CI) | 89.6 (87.7-91.3) | 81.8 (79.0-84.3) | 75.5 (72.3-78.3) | 69.1 (64.3-73.3) | 61.0 (53.3-67.4) | 47.2 (36.2-56.3) | 27.4 (6.0-43.9) | |
| **CoronaVac** |  |  |  |  |  |  |  | |
| Case (n_u_/n_v_) | 18766 / 369 | 18702 / 513 | 18779 / 712 | 18417 / 536 | 18206 / 347 | 18003 / 343 | 17860 / 144 | |
| Control (n_u_/n_v_) | 58272 / 3579 | 58565 / 3303 | 58989 / 3597 | 58490 / 2139 | 58084 / 1347 | 57977 / 781 | 57622 / 293 | |
| VE (95% CI) | 71.7 (68.0-74.9) | 62.5 (58.1-66.4) | 55.2 (50.6-59.3) | 44.8 (38.2-50.7) | 45.5 (37.2-52.6) | 5.3 (-11.3-19.5) | -6.2 (-36.9-17.5) | |
| *COVID-19 related mortality* | | | | | | | |  |
| **BNT162b2** |  |  |  |  |  |  |  | |
| Case (n_u_/n_v_) | 5935 / 5 | 5920 / 4 | 5926 / 6 | 5865 / 7 | 5824 / 3 | 5806 / 3 | 5781 / 3 | |
| Control (n_u_/n_v_) | 21171 / 787 | 21224 / 598 | 21192 / 494 | 21197 / 242 | 21165 / 113 | 21158 / 46 | 21142 / 13 | |
| VE (95% CI) | 94.5 (84.8-98.0) | 95.2 (82.3-98.7) | 94.8 (81.5-98.5) | 94.2 (73.9-98.7) | 96.9 (72.8-99.6) | -196.8 (-4067.4-78.9) |  | |
| **CoronaVac** |  |  |  |  |  |  |  | |
| Case (n_u_/n_v_) | 5935 / 8 | 5920 / 31 | 5926 / 29 | 5865 / 16 | 5824 / 12 | 5806 / 7 | 5781 / 1 | |
| Control (n_u_/n_v_) | 21171 / 951 | 21224 / 770 | 21192 / 646 | 21197 / 370 | 21165 / 240 | 21158 / 101 | 21142 / 15 | |
| VE (95% CI) | 93.8 (83.8-97.6) | 78.0 (59.1-88.1) | 88.6 (73.9-95.0) | 80.2 (53.4-91.6) | 89.1 (66.9-96.4) | 76.1 (-37.6-95.8) | 57.9 (-425.3-96.6) | |
| *COVID-19 related severe complications* | | | | | | | |  |
| **BNT162b2** |  |  |  |  |  |  |  | |
| Case (n_u_/n_v_) | 1072 / 9 | 1077 / 9 | 1078 / 15 | 1037 / 11 | 1040 / 7 | 1015 / 7 | 1006 / 3 | |
| Control (n_u_/n_v_) | 3312 / 218 | 3323 / 200 | 3331 / 188 | 3326 / 85 | 3313 / 58 | 3301 / 36 | 3288 / 9 | |
| VE (95% CI) | 90.0 (78.4-95.4) | 90.6 (79.9-95.6) | 86.7 (74.2-93.2) | 76.7 (48.0-89.5) | 78.6 (46.8-91.4) | 56.1 (-17.4-83.6) | 75.1 (-37.7-95.5) | |
| **CoronaVac** |  |  |  |  |  |  |  | |
| Case (n_u_/n_v_) | 1072 / 8 | 1077 / 20 | 1078 / 24 | 1037 / 19 | 1040 / 14 | 1015 / 8 | 1006 / 5 | |
| Control (n_u_/n_v_) | 3312 / 222 | 3323 / 187 | 3331 / 209 | 3326 / 118 | 3313 / 105 | 3301 / 34 | 3288 / 3 | |
| VE (95% CI) | 91.1 (80.5-96.0) | 71.7 (50.9-83.7) | 75.9 (60.0-85.5) | 68.9 (43.1-83.0) | 69.9 (43.2-84.0) | 54.1 (-13.1-81.4) | 0.1 (-524.6-84.0) | |

n_u_: number of unvaccinated individuals; n_v_: number of vaccinated individuals whose time since last dose fell within the specific interval; VE: vaccine effectiveness; CI: confidence interval

**Supplementary Figure 1**. Trends of new COVID-19 cases, hospitalisation, ICU admission / ventilatory support and deaths during the study period

| (A) COVID-19 infection  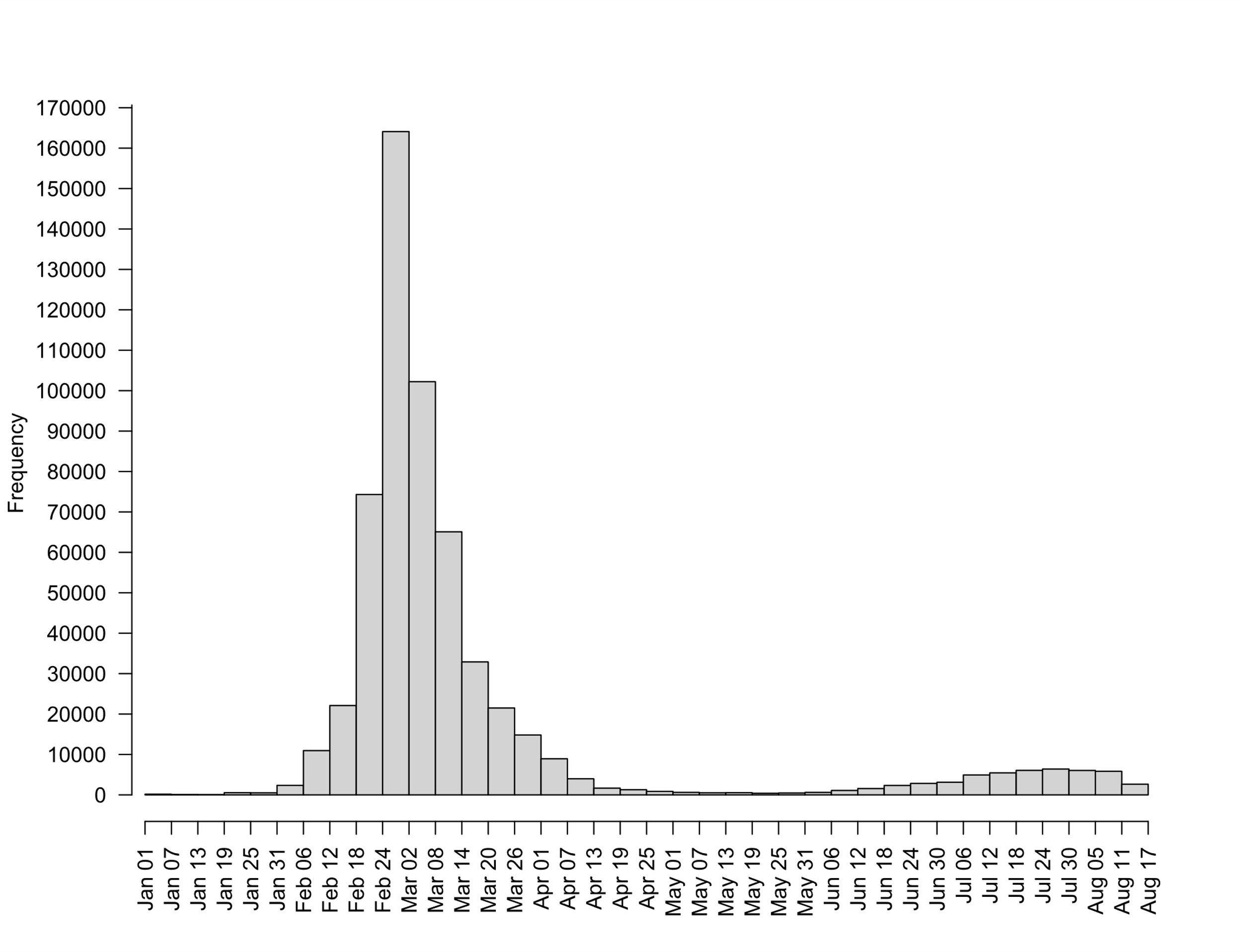 | (B) COVID-19-related hospitalisation  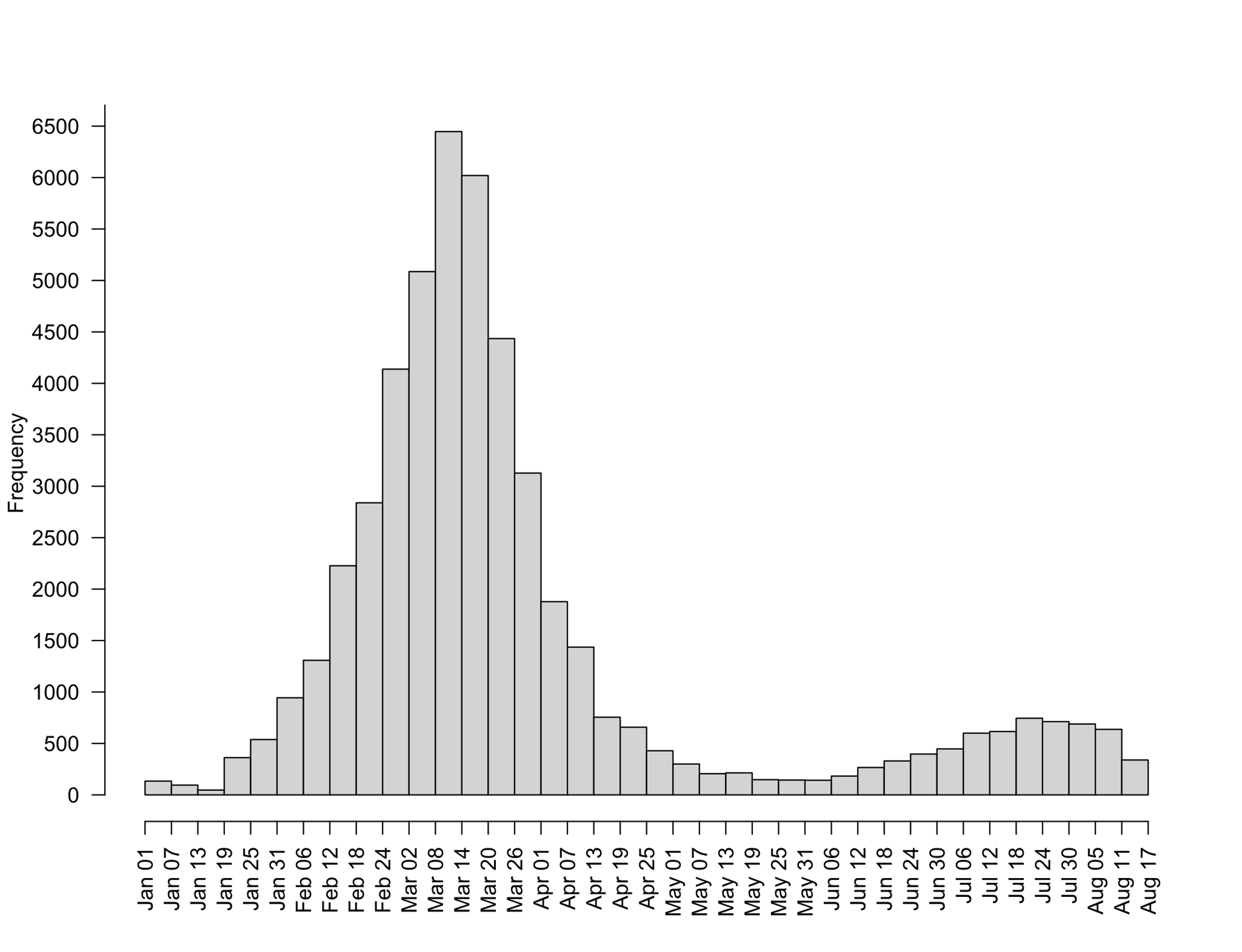 |
| --- | --- |
| (C) COVID-19-related severe complications  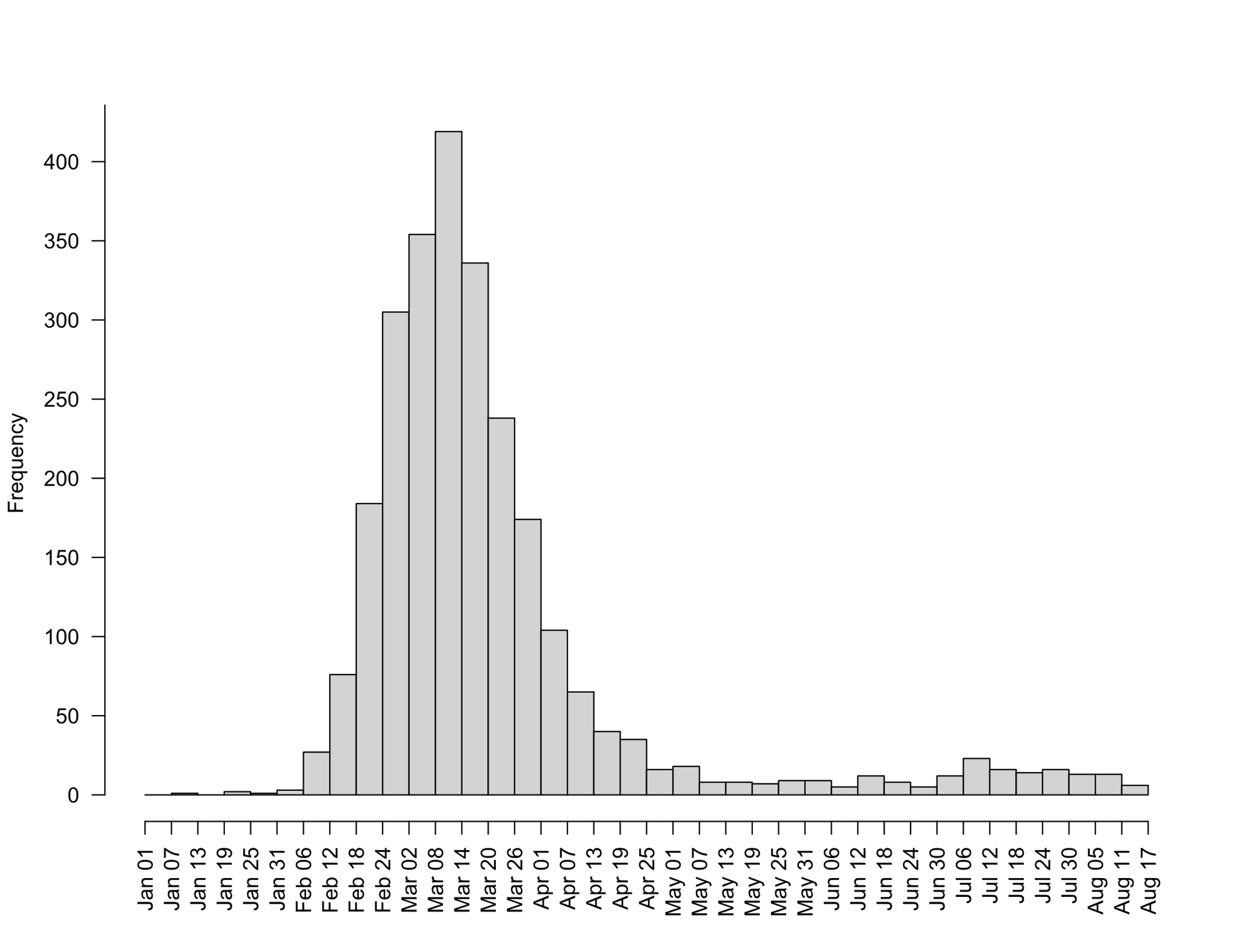 | (D) COVID-19-related deaths  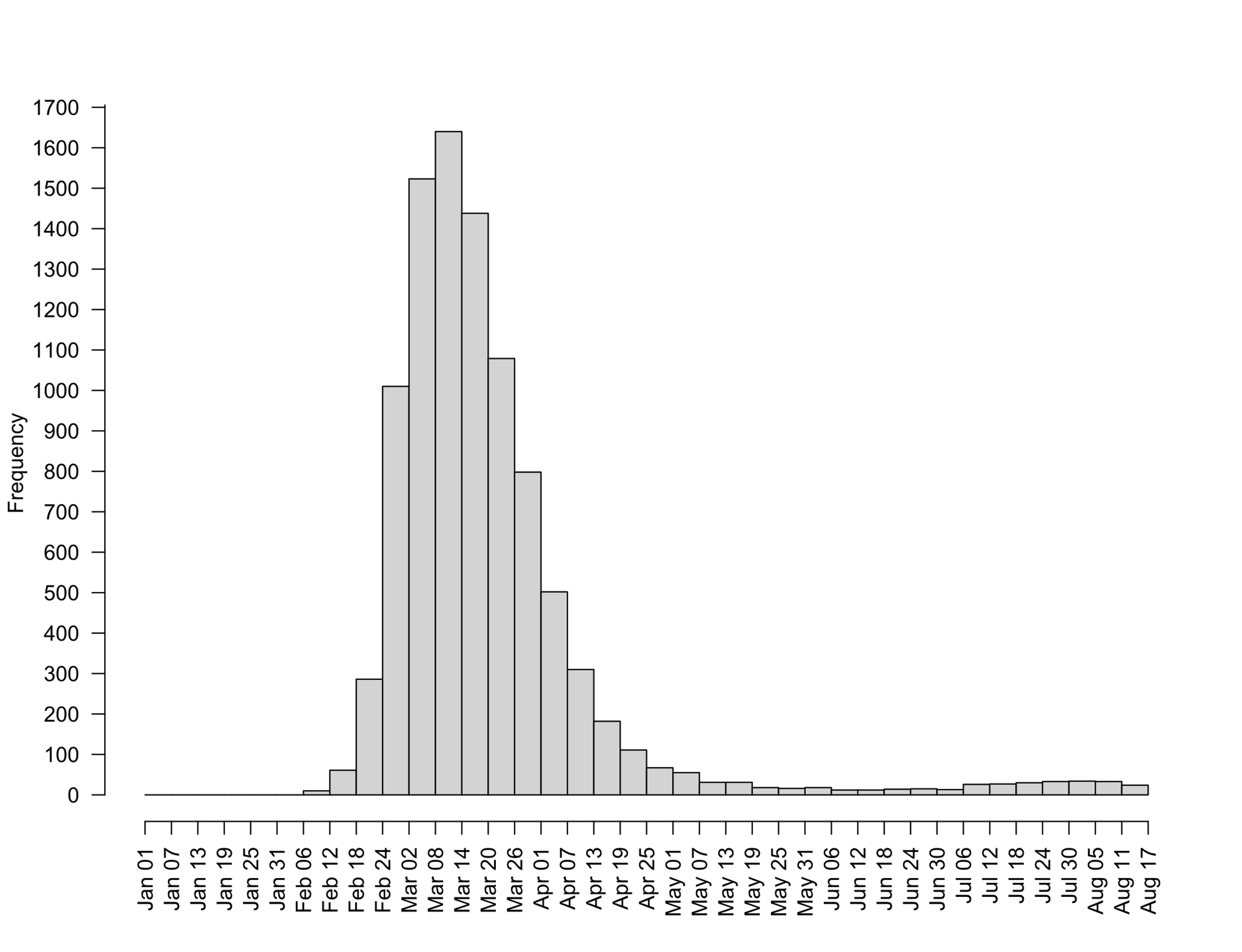 |

.

**Supplementary Figure 2.** Estimated rate of change in vaccine effectiveness after second or third dose BNT162b2 or CoronaVac vaccination


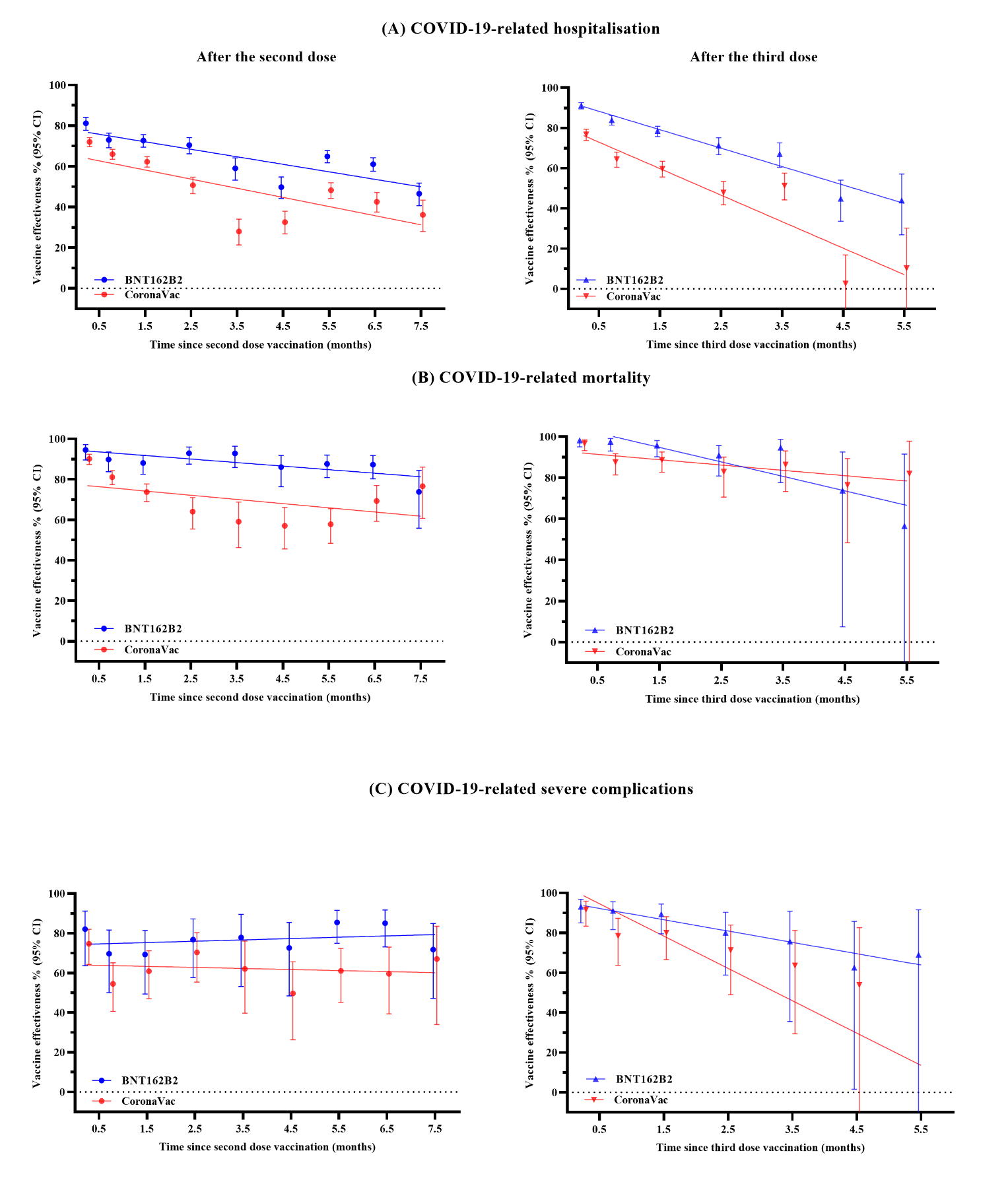


|  | **Estimated rate of change in VE (percentage points per month) (95% CI)** | | |
| --- | --- | --- | --- |
|  | **Hospitalisation** | **Mortality** | **Severe complications** |
| 2^nd^-dose |  |  |  |
| BNT162b2 | -3.69 (-5.85, -1.53) | -1.75 (-3.19, -0.32) | 0.67 (-1.45, 2.79) |
| CoronaVac | -4.49 (-8.06, -0.92) | -2.08 (-5.62, 1.46) | -0.53 (-3.15, 2.10) |
| 3^rd^-dose |  |  |  |
| BNT162b2 | -9.16 (-11.50, -6.82) | -7.06 (-11.50, -2.62) | -5.67 (-7.88, -3.47) |
| CoronaVac | -13.17 (-19.37, -6.97) | -2.60 (-4.79, -0.41) | -16.21 (-27.74, -4.68) |
